# A reciprocal feedback between colon cancer cells and Schwann cells promotes the proliferation and metastasis of colon cancer

**DOI:** 10.1186/s13046-022-02556-2

**Published:** 2022-12-15

**Authors:** Shengbo Han, Decai Wang, Yan Huang, Zhu Zeng, Peng Xu, Hewei Xiong, Zunxiang Ke, Ya Zhang, Yuhang Hu, Fan Wang, Jie Wang, Yong Zhao, Wenfeng Zhuo, Gang Zhao

**Affiliations:** Department of Emergency Surgery, Union Hospital, Tongji Medical College, Huazhong University of Science and Technology, Wuhan, 430022 China

**Keywords:** Schwann cells, Colon cancer, Metastasis, Exosomes, NGF, EMT

## Abstract

**Background:**

Research has indicated that the emergence of Schwann cells around premalignant lesions of colon cancer might be an early indicator promoting the onset of tumorigenesis. The present study explored the communication between colon cancer cells and Schwann cells.

**Methods:**

Immunofluorescence analyses were conducted to examine the differential distribution of Schwann cells within colon cancer tissues and normal colon tissues. CCK8 assay, colony formation assay, wound healing assay, and transwell assay were performed to investigate the interaction between colon cancer cells and Schwann cells. Exosomes derived from colon cancer cells were isolated to further explore the effect of colon cancer cells on Schwann cells. Gain- and loss-of function experiments, luciferase reporter assays, chromatin immunoprecipitation assays, and immunohistochemistry assays were performed to reveal the cross-talk between colon cancer cells and Schwann cells. Furthermore, colon cancer cells co-cultured with Schwann cells were transplanted into nude mice for evaluating their effect on tumor proliferation and metastasis in vivo.

**Results:**

The clinicopathological characteristics indicated that Schwann cells were enriched in colon cancer tissues and were associated with tumor metastasis and poor prognosis. The co-culture of Schwann cells with colon cancer cells promoted the proliferation and migration of colon cancer cells and Schwann cells, which was mediated by nerve growth factor (NGF) secreted from Schwann cells. Exosomal miR-21-5p released by colon cancer cells inhibited VHL expression in Schwann cells, which in turn stabilized the HIF-1α protein and increased the transcription of NGF. Meanwhile, the Schwann cells-derived NGF activated TrkA/ERK/ELK1/ZEB1 signaling pathway in colon cancer cells, which further enhanced the expression of exosomal miR-21-5p. Inhibition of either NGF or miR-21-5p significantly inhibited the proliferation and metastasis of transplanted colon cancer cells in nude mice. Coincidently, miR-21-5p was positively associated with the expression of NGF, p-ERK, p-ELK1, and ZEB1 in human colon cancer tissues.

**Conclusions:**

Our results implicated a reciprocal communication between colon cancer cells and Schwan cells that promoted the proliferation and metastasis of colon cancer, and identified NGF and exosomal miR-21-5p as potential therapeutic targets for the treatment of colon cancer.

**Supplementary Information:**

The online version contains supplementary material available at 10.1186/s13046-022-02556-2.

## Background

Colon cancer ranks fifth in terms of the cancer lethality and incidence globally, and nearly 1.2 million new patients and 0.6 million deaths were associated with colon cancer in 2020 [1]. The survival rate of colorectal cancer (CRC) patients with a localized disease was 90% at 5 years. However, the survival rate of CRC patients with regional or distant diseases was reduced to 71 and 14%, respectively [2]. The tumor microenvironment (TME) consisting of cancer-associated fibroblasts, vascular cells, and infiltrating immune cells, regulates the proliferation, death, and metastasis of tumor cells in a cell non-autonomous manner [3]. Hence, targeting the TME may be an effective strategy for treating colon cancer.

Among the components of the TME, the peripheral nervous system (PNS) has been reported to be involved in the progression and dissemination of various types of cancers [4]. Schwann cells, which are the major glial cells in the PNS, have been shown to participate in the spreading and metastasis of lung and pancreatic cancer through direct contact or paracrine manner [5, 6]. Perineural invasion (PNI) is associated with more aggressive tumors and poorer prognosis in various tumors, especially in head and neck cancer, prostate cancers, and CRC [7–9]. Demir et.al reported that Schwann cells migrated to colon cancer cells, rather than normal colon cells, before the onset of tumor invasion into peripheral nerves [10]. Nevertheless, it is still unclear whether Schwann cells facilitated the progression and metastasis of colon cancer.

Epithelial-mesenchymal transition (EMT) is a feature of transformed epithelial cells that undergo loss of cellular polarity and gain mesenchymal characteristics in solid tumors [11]. EMT was thought to be a major driver of tumors from initiation to metastasis [12–14]. It was shown that EMT participated in the Schwann cell-induced metastasis of salivary adenoid cystic carcinoma, and pancreatic and lung cancer [5, 6, 15]. Schwann cells activated by pancreatic cancer cells released IL6, which activated STAT3 signaling and induced the EMT process in pancreatic cancer cells, thereby promoting the metastasis of pancreatic cancer cells [6]. Hence, further studies are needed to explore the precise role of EMT in mediating the interaction between Schwann cells and colon cancer cells.

Herein, we explored the correlation of Schwann cell invasion with metastasis and poor prognosis of colon cancer. Schwann cells facilitated the proliferation, invasion, EMT, and metastasis of colon cancer cells. Furthermore, colon cancer cells augmented NGF expression in Schwann cells through exosomes, forming a positive feedback loop. Finally, blocking this reciprocal feedback attenuated Schwann cell-induced progression of colon cancer, revealing potential therapeutic targets for colon cancer treatment. However, further studies are required to unravel the molecular mechanisms underlying the interaction between Schwann cells and colon cancer cells.

## Methods

### Patients and clinical specimens

Colon cancer tissues were acquired from sixty-four patients who underwent radical surgery at Wuhan Union Hospital between June 2015 and July 2018. None of the patients underwent chemoradiotherapy before surgery. Each patient signed an informed consent form before the sample was obtained. This study was approved by the Human Research Ethics Committee of Hua Zhong University of Science and Technology.

### Co-culture system and conditioned medium preparation

For the co-culture assay, we used a 0.4 μm pore Transwell chamber (Corning, USA). 4 × 10^5^ colon cancer cells (SW480, HCT116) were plated in the bottom chamber of 6-well plates, while 4 × 10^5^ Schwann cells (sNF96.2) were added into the upper Transwell insert. The cells were co-cultured for 48 hours and were used for subsequent experiments. To collect the conditioned medium (CM) of FHC, SW480, and HCT116 cells, 4 × 10^5^ cells were plated in 6-well plates and cultured in DMEM/F12 or DMEM/high glucose for 48 h. The supernatant was collected and centrifuged it at 2000×g for 10 minutes to eliminate the cells and cell debris. All the CMs were used instantly or frozen at − 80 °C.

### qRT-PCR

Total RNA was extracted from cells or frozen tissues according to the manufacturer’s instructions for Trizol (TaKaRa, Japan) reagent. Then, cDNA was reverse transcribed by RT Master Mix (TaKaRa, Japan). RT-PCR was conducted using the SYBR Master Mix (TaKaRa, Japan) on Applied Biosystems StepOne-Plus system (ABI, USA), based on the manufacturer’s instructiosn. The expression levels of cellular RNA and mRNA were normalized against the housekeeping gene GAPDH. U6 served as a control for miRNA. The primer sequences are listed in Supplementary Table S2.

### Western blotting

We used RIPA buffer containing PMSF (Sigma, USA) and phosphorylase inhibitors (Sigma, USA) to lyse the cells and tissues. Cell extracts were centrifuged at 12000×g for 10 minutes at 4 °C. Firstly, protein specimens were separated based on their molecular weight by SDS-PAGE. Then, target proteins were transferred to PVDF membranes (Millipore, USA). Then, the PVDF membranes were incubated with the respective primary antibodies (Table S3) for overnight at 4 °C. This was followed by incubation with the secondary antibody (CST, USA) for 1-hour the subsequent day. The protein bands were visualized using ECL (Pierce, USA), and collected by ChemiDocTm XRS Molecular Imager system (Bio-Rad, USA). Finally, the Image J software was used to analyze the band densities.

### Transfection assay

All the overexpression plasmids targeting VHL (pcDNA3.1-VHL), HIF-1α (pcDNA3.1-HIF-1α), and ZEB1 (pcDNA3.1-ZEB1) were designed and synthesized by GeneChem (China), and the empty plasmid was used as a negative control. HIF-1α siRNA (siHIF-1α), VHL siRNA (siVHL), ELK1 siRNA (siELK1), ZEB1 siRNA (siZEB1), and corresponding negative control were purchased from RiboBio (Guangzhou, China). MiR-21-5p mimics, miR-21-5p inhibitors, and matched control (mimic-NC or inhibitor-NC) were synthesized by Invitrogen (Shanghai, China). All the small interference RNAs were transfected at a final concentration of 50 nM, and the plasmid was transfected at a final concentration of 0.2 μg for 96 well plates and 1.6 μg for 12 well plates. Lipofectamine 3000 (Invitrogen, USA) was used for cell transfections according to the manufacturer’s instructions. Protein and total RNA were extracted after 48 h.

### Transwell migration and invasion assays

8 × 10^4^ cells in medium without FBS were plated in an 8 μm pore Transwell chamber (BD, USA) coated with 40 μl Matrigel (BD, USA) for invasion assays or into uncoated chamber for the migration assays. Culture medium supplemented with 10% FBS was placed in the lower chambers. Then, the cells were gently wiped away on the top of the filters after incubation for 24 hours for the migration assays and after 30 hours for the invasion assays. The cells were fixed on the membranes with 5% paraformaldehyde for 20 minutes followed by staining for 15 minutes with 0.1% crystal violet. Lastly, five fields of vision were chosen and the number of cells were calculated under the microscope.

### Wound healing assays

Cells were plated in 6-well plates overnight. 200 μL pipette tips were used to scratch a straight line when the cells achieved 60-80% confluence. Cells were then cultured with medium without FBS. Then, a picture of the cell wound width was taken under the microscope at 0, 24, and 48 hours.

### Exosome isolation and treatment

Cells were cultured in medium with exosome-free serum to remove the interference of serum exosomes. Briefly, the serum was centrifuged at 100,000×g for more than 16 hours, and filtered with a 0.22 μm filter (Millipore, USA). The medium was collected after 24-72 hours, and the exosomes were isolated according to the instructions on the kit (Invitrogen, California, USA). The medium was centrifuged at 2000×g for 30 min to eliminate the cells and debris. The total exosome isolation reagent was added to it and incubated between 2 °C to 8 °C overnight. The exosomes were centrifuged at 10000×g for 1 h and resuspended in PBS. qNano and electron microscope were used to quantify the size and concentration of the exosomes and to visualize the morphology of the exosomes. Finally, the exosomes were labeled with PKH26 and were extracted again in the reagent. Exosomes were isolated from 4 × 10^5^ FHC or colon cancer cells. Schwann cells were plated on 12-well plates the day before treatment. 100 μg exosomes were added to the plates when the Schwann cells reached 70% confluence. The cells were collected after 48 h for the subsequent experiments.

### Immunohistochemistry (IHC)

Briefly, tissues were formalin-fixed, dehydrated, and paraffin-embedded. Then, the tissue sections were incubated with primary antibodies overnight at 4 °C. Next day, the tissue sections were incubated with HRP-conjugated secondary antibodies for 1 hour at 37 °C. Then sections were further washed with PBS and distilled water, freshly prepared DAB solution (diaminobenzidine) was subsequently used until the tissue sections were ready to observe. On the one hand, we evaluated the intensity of tissue staining. We calculated the percentage of positive tissue staining (graded as 0, < 5%; 1, 5-25%; 2, 26-50%; 3, 51-75%; and 4, > 75%). SI score was equal to the product of those two. Two experienced pathologists evaluated all the results from the IHC analysis of the tissue sections.

### Immunofluorescence (IF)

SW480 and HCT116 cells were placed on coverslips and fixed with 5% paraformaldehyde when the cells achieved 60-70% confluence. The cells were blocked with 5% donkey serum for 1 hour and incubated with primary antibodies overnight at 4 °C. Antibodies for IF assays are listed in Supplementary Table 2. The next day, the cells were incubated with the corresponding CY3 secondary antibody (Jackson Immuno Research, Ely, British) for 1 hour at 37 °C. After counterstaining the nuclei with DAPI (Sigma, St Louis, MO, USA) for 15 minutes, the fluorescent images were captured by epifluorescence microscopy (Olympus, Tokyo, Japan).

### Statistical analysis

All data were analyzed by GraphPad Prism5.0 and all assays were repeated at least three times. We used t-test to analyze the frequency of Schwann cells in colon cancer tissues and paired adjacent normal tissues. Pearson’s correlation analysis was performed to analyze the correlation between NGF and ZEB1. We used the *chi*-square test to identify the correlation between miR-21-5p and NGF, p-ERK, p-ELK1, and ZEB1 in the colon cancer specimens. χ2 test was used to analyze the relationship between Schwann cell frequency and the clinical features of colon cancer. *P* < 0.05 was considered to be statistically significant and all tests were two-sided.

## Results

### Schwann cells are enriched in colon cancer tissues and associated with metastasis and poor prognosis of colon cancer patients

**Fig. 1:**
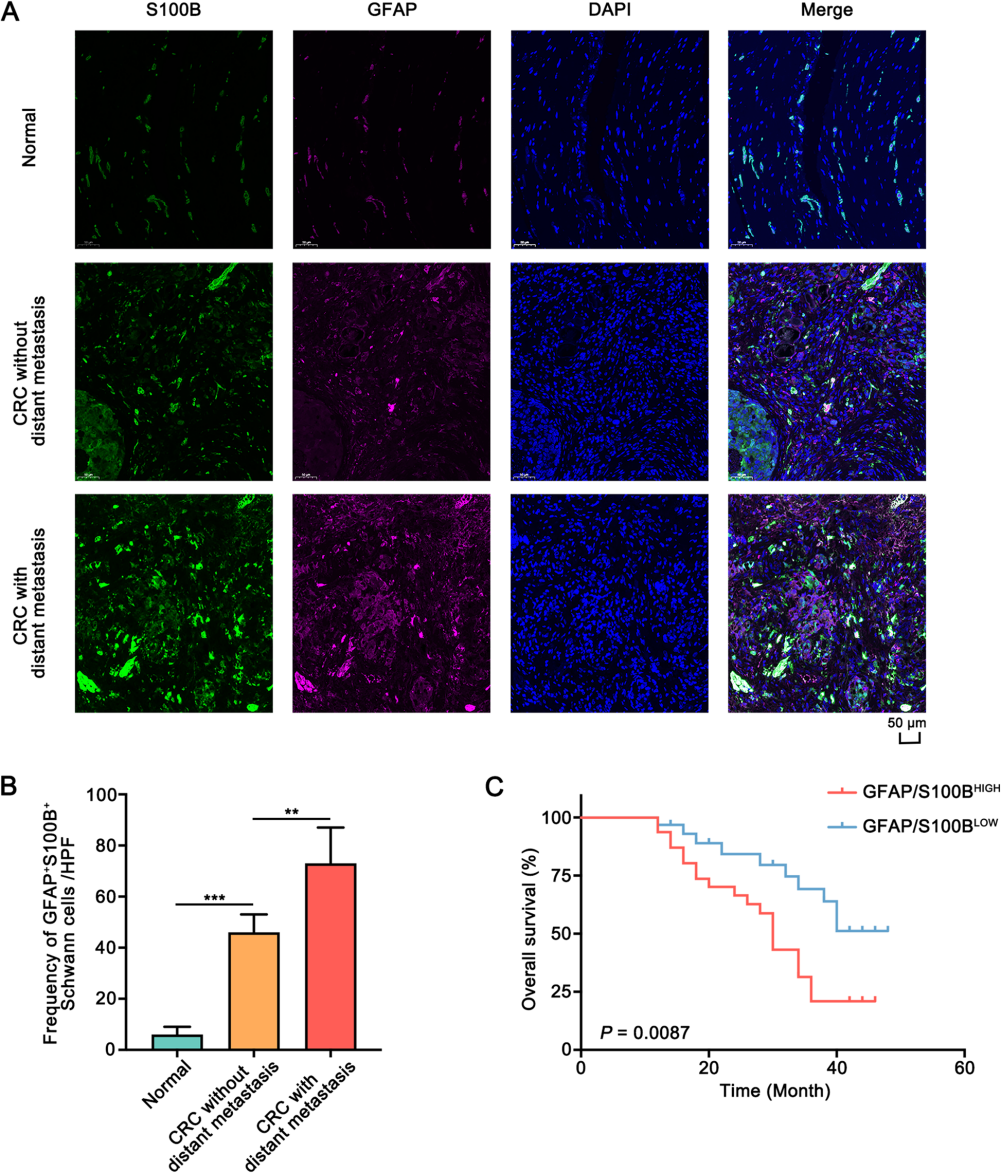
The distribution of Schwann cells in colon cancer and normal colorectal tissues.** A**. Double immunofluorescence images showed that Schwann cells were relatively scarce in normal colorectal tissues (upper panel), while were abundant in colon cancer tissue (middle panel, lower panel). Magnification, × 200. **B**. The presence of Schwann cells in patients with distant metastasis was more abundant than that in patients without distant metastasis. The *chi*-squared test was used to assess the difference. **C**. Kaplan-Meier survival analysis indicated that the colon cancer patients in the GFAP^+^/S100B^+^-HIGH group had a shorter overall survival time. All data were revealed as means ± standard deviation (SD) for no less than three independent experiments. Significant *P* values showed as **P < 0.01, and ***P < 0.001

To determine the presence of Schwann cells in colon cancer tissues, double immunofluorescence (IF) assay was performed in sixty-four pairs of colon cancer tissues and adjacent normal tissues. S100B and glial-fibrillary-acidic protein (GFAP) are the traditional markers of Schwann cells. The results showed that Schwann cells were mainly located in colon cancer tissues and were rare in the adjacent normal tissues (Fig. 1A, B). Furthermore, the frequency of Schwann cells in patients with distant metastasis was higher than in patients without distant metastasis (Fig. 1A, B). Moreover, the clinicopathological characteristics of colon cancer patients showed that the high frequency of Schwann cells was positively associated with the T stage, N stage, and M stage (Table S1). Additionally, Kaplan-Meier survival analysis indicated that colon cancer patients in the GFAP/S100B-HIGH group had a shorter overall survival time than those in the GFAP/S100B-LOW group (Fig. 1C). Taken together, Schwann cells were enriched in colon cancer tissues and were associated with metastasis and poor prognosis of colon cancer patients.

### Colon cancer cells boost the proliferation and migration of Schwann cells by stimulating NGF secretion from Schwann cells

**Fig. 2:**
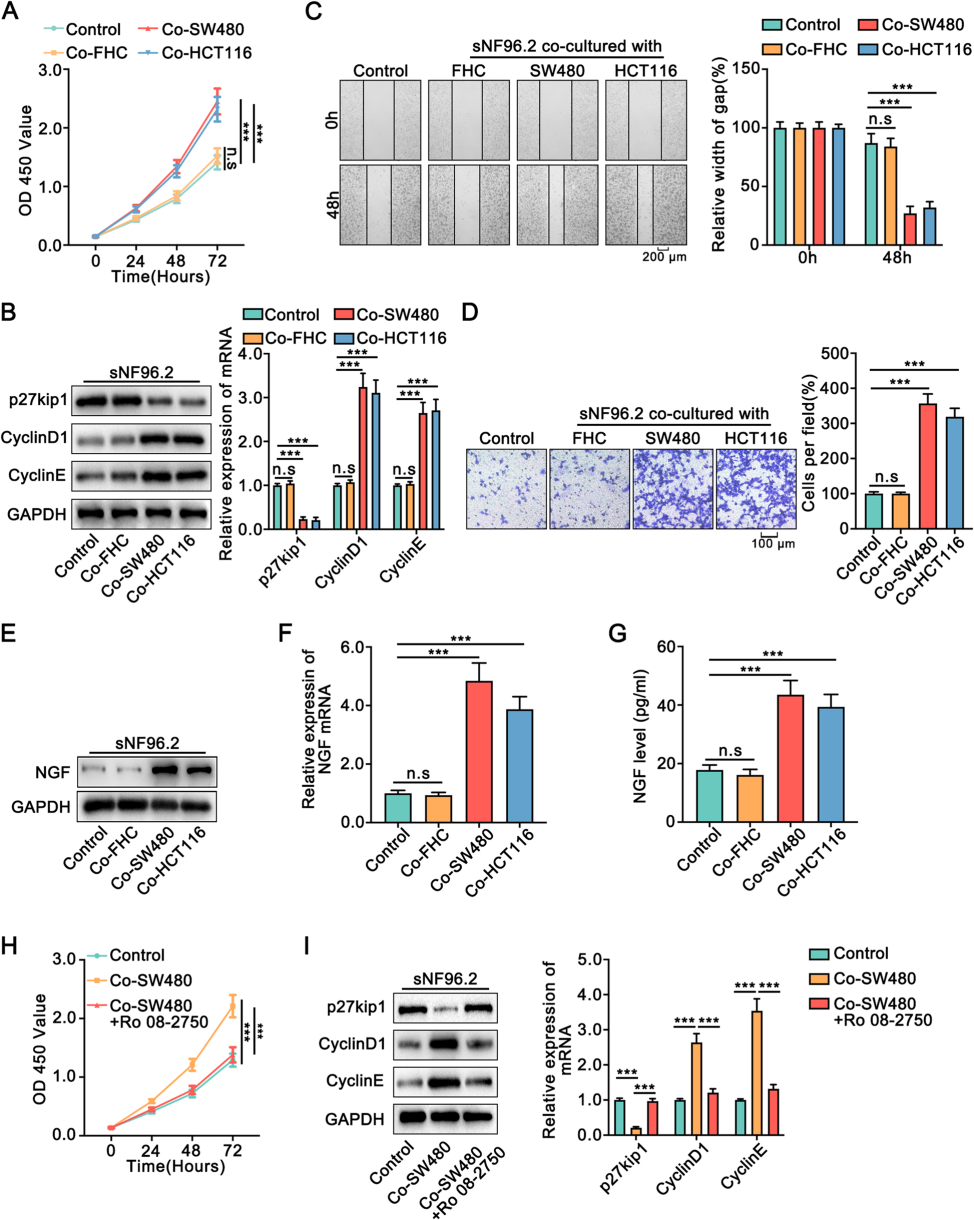
Colon cancer cells promoted the proliferation and migration of Schwann cells by stimulating their secretion of NGF.** A**. The proliferation of Schwan cells co-cultured with FHC, SW480, and HCT116 cells or not was assessed via CCK8 for 3 days. **B**. The expression of p27kip1, CyclinD1, and CyclinE of Schwann cells co-cultured with FHC, SW480, and HCT116 cells or not was detected by Western blot and qRT-PCR. **C**. The wound healing assays showed that co-cultured with colon cancer cells significantly augmented the migrative ability of Schwann cells. **D**. The transwell assays indicated that co-cultured with colon cancer cells increased the migrative abilities of Schwann cells. **E, F**. The expression of NGF in Schwann cells was increased when co-cultured with colon cancer cells by Western blot and qRT-PCR. **G**. ELISA assay showed the expression of NGF in Schwann cells was augmented when co-cultured with colon cancer cells. **H**. CCK8 assay showed that inhibition of NGF reversed the enhanced proliferation of Schwann cells co-cultured with SW480 cells. **I**. Western blot and qRT-PCR showed that inhibition of NGF blocked the altered expression of p27kip1, CyclinD1, and CyclinE in Schwann cells that co-cultured with SW480 cells. Ro 08-2750 :the inhibitor of NGF. All data were revealed as means ± standard deviation (SD) for no less than three independent experiments. Significant *P* values showed as ***P < 0.001. n.s means the difference was not significant

Previously, it was reported that Schwann cells migrated to colon cancer cells rather than normal colon cells, before the onset of tumor invasion by peripheral nerves [10]. To evaluate the cross-talk between colon cancer cells and Schwann cells, we co-cultured Schwann cells with colon cancer cells. The CCK8 assay results showed that the co-culture of SW480 cells and HCT116 cells significantly increased the OD450 value of Schwann cells (Fig. 2A). Furthermore, colon cancer cells augmented the expression of CyclinD1 and CyclinE (vital cell proliferation regulatory proteins) and attenuated the expression of p27kip1 (the cell cycle inhibitor) in Schwann cells (Fig. 2B). Next, we explored the role of colon cancer cells in the migration of Schwann cells. Wound healing assays and transwell assays showed that colon cancer cells increased the migration of Schwann cells (Fig. 2C, D).

Schwann cells, the most important glial cells in the PNS, regulate the expression of various neurotrophic factors including NGF [16]. These results inspired us to investigate the potential mechanism of how colon cancer cells induced the proliferation and migration of Schwann cells. Moreover, it was reported that NGF promoted the proliferation and migration of Schwann cells [17, 18]. Previous studies showed that co-culture of Schwann cells with pancreatic cancer cells increased NGF production by Schwann cells [10]. Similarly, we found that NGF expression was elevated in Schwann cells both at mRNA and protein levels upon co-culture with colon cancer cells (Fig. 2E, F). ELISA assays indicated that the secretion of NGF was augmented in Schwann cells upon co-culture with colon cancer cells (Fig. 2G). Moreover, blocking NGF significantly reversed the effect of colon cancer cells on the proliferative and migratory ability of Schwann cells (Fig. 2H, I, S1A, B). Also, upon the addition of recombinant human NGF to Schwann cells at the same concentration as that secreted by Schwann cells in co-culture with SW480 cells showed that NGF strengthened the proliferative and migratory abilities of Schwann cells (Fig. S1C-F). In conclusion, our data demonstrated that colon cancer cells enhanced the proliferation and migration of Schwann cells by stimulating NGF secretion from Schwann cells.

### Exosomal miR-21-5p from colon cancer cells promoted NGF expression in Schwann cells

**Fig. 3:**
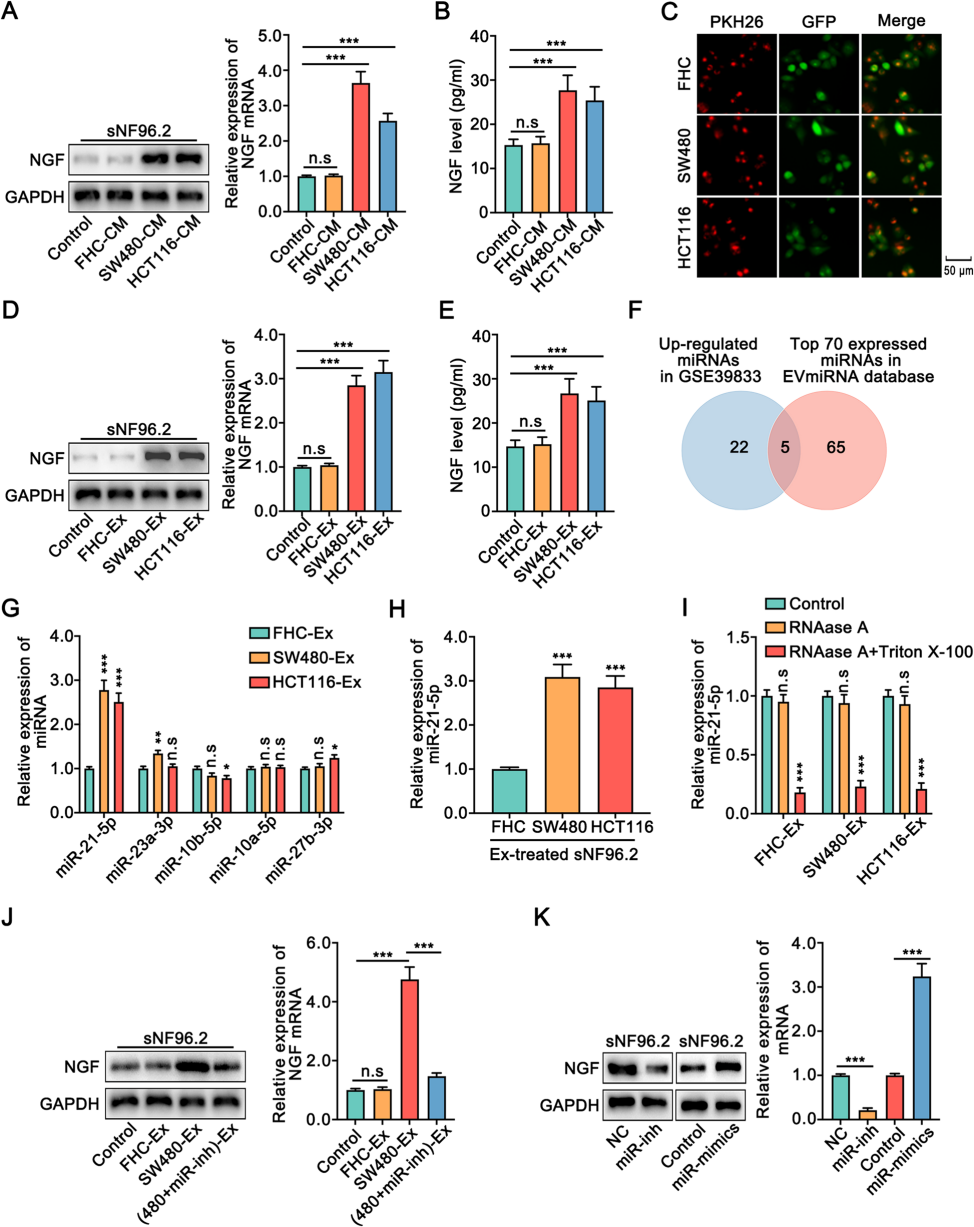
Exosomes derived from colon cancer cells facilitated the expression of NGF in Schwann cells via miR-21-5p.** A**. The expression of NGF in Schwann cells was increased when added colon cancer-CM was by Western blot and qRT-PCR**. B**. ELISA assay showed that the expression of NGF in Schwann cells was augmented when colon cancer-CM was added. **C.** Exosomes were labeled with PKH26, and sNF96.2 cells were transfected lentivirus with stable expression of GFP. Incubation of sNF96.2 cells with PKH26-labeled exosomes for 6 h. **D.** Western blot and qRT-PCR showed that the expression of NGF in Schwann cells was increased when added colon cancer-Ex. **E.** ELISA assay showed the expression of NGF in Schwann cells was augmented when added colon cancer-Ex. **F.** Venn diagram indicated that five miRNAs were highly expressed in exosomes of colon cancer serum and tissues from the GEO and EVmiRNA database. **G.** The qRT-PCR showed the expression of five miRNAs in the exosomes of FHC, SW480, and HCT116 cells. **H.** The qRT-PCR showed the expression of miR-21-5p in sNF96.2 cells treated with exosomes. **I.** The qRT-PCR analysis of miR-21-5p in the CM of FHC, SW480, and HCT116 cells was treated with RNase R (3 U/μg) alone or combined with Triton X-100 (0.1%) for 20 min. **J.** Western blot and qRT-PCR showed that inhibition of miR-21-5p in SW480 cells significantly blocked the increased expression of NGF in Schwann cells incubated with the exosome of the SW480 cells. **K.** Western blot and qRT-PCR showed that the expression of NGF was conspicuously increased or decreased in Schwann cells upon miR-21-5p overexpression or knockdown in Schwann cells, respectively. CM: conditioned medium. Ex: Exosomes. Inh: inhibitor. All data were revealed as means ± standard deviation (SD) for no less than three independent experiments. Significant *P* values showed as *P < 0.05, **P < 0.01, ***P < 0.001. n.s means the difference was not significant

The expression and secretion of NGF in Schwann cells were elevated upon co-culture with colon cancer cells (Fig. 2E-G). Coincidentally, the expression and secretion of NGF were increased in Schwann cells treated with conditioned medium from SW480 or HCT116 cells, however, there was no significant change in Schwann cells treated with conditioned medium from FHC cells (Fig. 3A, B). It has been reported that tumor cells secrete more exosomes than normal cells and exosomes play an important role in intercellular communication [19, 20]. Thus, we hypothesized that colon cancer cells might facilitate the secretion of NGF in Schwann cells through exosomes. Exosomes were labeled with the fluorescent dye PKH26, and our data showed that exosomes derived from FHC, SW480, and HCT116 cells were fused to Schwann cells (Fig. 3C). The exosomal marker CD63 was bound, while the golgi compartment-specific marker GM130, was absent in exosomes (Fig. S2B). Western blot, qRT-PCR, and ELISA assays showed that NGF expression was increased in Schwann cells when incubated with exosomes from colon cancer cells (Fig. 3D, E).

miRNAs are the most abundant cargo in exosomes and are involved in intercellular communication [21, 22]. To explore how exosomes from colon cancer cells promoted the expression of NGF in Schwann cells, we analyzed the miRNA sequencing data of exosomes from the GEO database. Twenty-seven miRNAs were upregulated in the serum of colon cancer patients as compared to healthy controls (Fig. 3F, S2A). Among these miRNAs, miR-21-5p, miR-23a-3p, miR-10b-5p, miR-10a-5p, and miR-27b-3p were also highly expressed in the colon cancer exosomes from EVmiRNA database (Fig. 3F, S2A). Furthermore, both intracellular and exosomal miR-21-5p expression levels were significantly elevated in colon cancer cells compared to FHC cells (Fig. 3G, S2C). Meanwhile, the expression of miR-21-5p in Schwann cells was increased upon incubation with exosomes from colon cancer cells (Fig. 3H). However, pre-miR-21-5p expression showed no significant change in Schwann cells upon treatment with colon cancer cell-derived exosomes, indicating that the transcriptional activity of miR-21-5p was not changed in Schwann cells and that the upregulated expression miR-21-5p in Schwann cells was caused mediated through exosomes (Fig. S2D). Moreover, treatment with RNAase A and Triton X-100 decreased miR-21-5p expression in exosomes, but RNAase A alone had no significant effect (Fig. 3I). Also, inhibition of miR-21-5p in colon cancer cells suppressed the exosome mediated increase in NGF expression in Schwann cells (Fig. 3J, S2E). The expression of NGF was obviously increased or decreased upon miR-21-5p overexpression or inhibition in Schwann cells, respectively (Fig. 3K). Collectively, our data indicated that exosomal miR-21-5p derived from colon cancer cells induced the expression of NGF in Schwann cells.

### Exosomal miR-21-5p facilitates NGF expression in Schwann cells through VHL/ HIF-1α axis

**Fig. 4:**
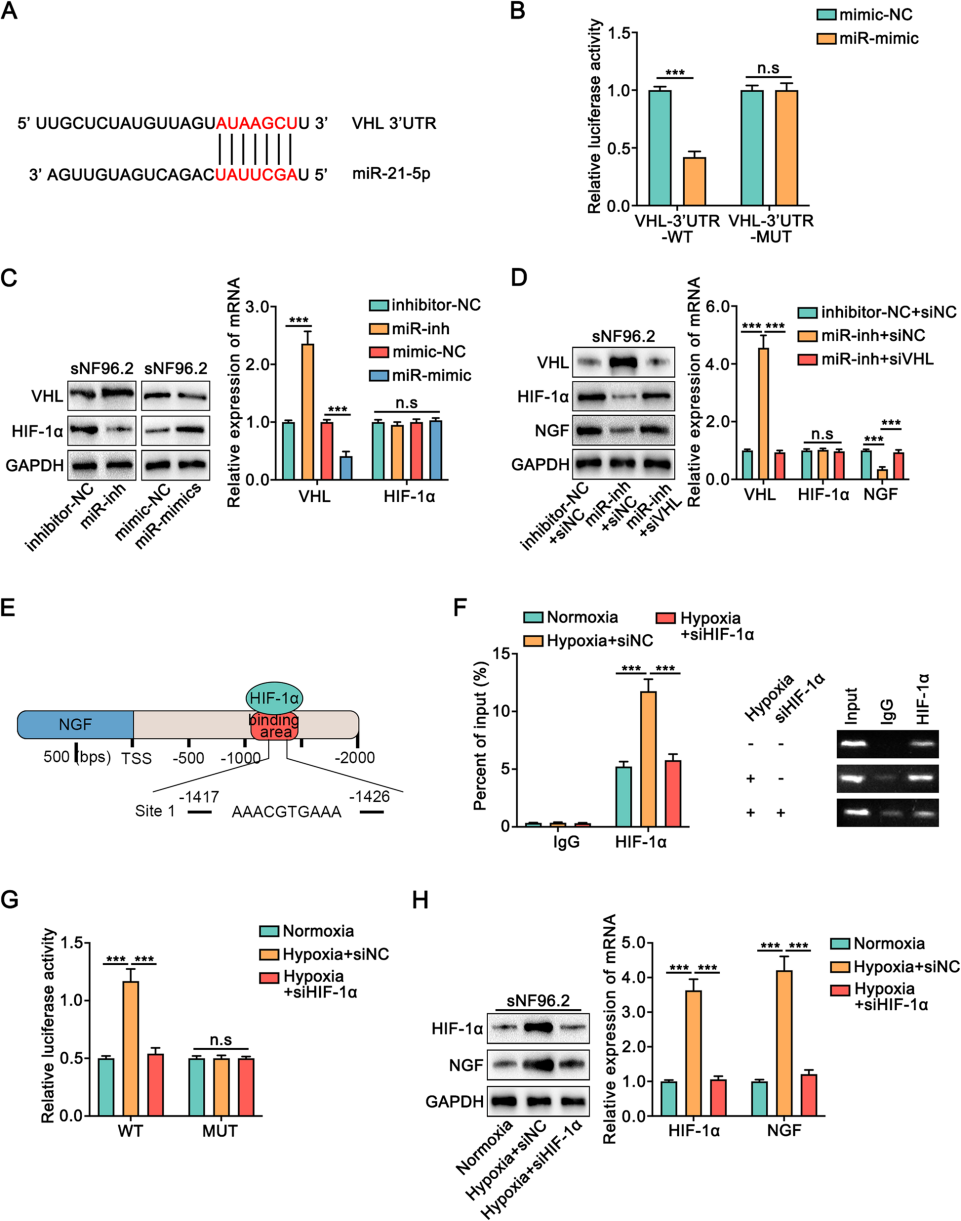
miR-21-5p promoted the expression of NGF in Schwann cells through VHL/HIF-1α.** A.** A miR-21-5p binding site of on the 3′-UTR of the VHL mRNA was predicted by TargetScan database. **B.** The binding of miR-21-5p on VHL 3′ UTR was evaluated by dual luciferase reporter assay. sNF96.2 cells were transfected with reporter plasmid containing wild type VHL 3′ UTR (WT) or mutant type (MUT) respectively, followed by transfection with miR-21-5p mimic (miR-mimic) or negative control (mimic-NC). **C.** sNF96.2 cells were transfected with mimic or inhibitor for miR-21-5p (miR-mimic, miR-inh), as well as associated negative control (miR-NC, inhibitor-NC). The regulation of miR-21-5p on the expression of VHL and downstream target HIF-1α was detected by Western blot and qRT-PCR assay. **D.** sNF96.2 cells were co-transfected with miR-inh and siRNA for VHL (siVHL), and associated negative control (inhibitor-NC, siNC). The regulation of miR-21-5p/VHL on HIF-1α/NGF expression was measured by Western blot and qRT-PCR assay. **E.** The schematic diagram exhibited one predicted binding site between HIF-1α and the promoter of NGF. **F.** sNF96.2 cells were transfected with siRNA for HIF-1α (siHIF-1α) or negative control (siNC), and further cultured under normoxia or hypoxia condition. The binding of HIF-1α on promoter of NGF was verified by ChIP assay by using anti-HIF-1α antibody or IgG. **G.** The activity of HIF-1α on NGF promoter was evaluated by dual luciferase reporter assay. sNF96.2 cells were transfected with dual luciferase reporter plasmid containing wild type (WT) or mutant type (MUT) promoter, which was further transfected with siHIF-1α or siNC and cultured under normoxia or hypoxia condition. **H.** sNF96.2 cells were transfected with siHIF-1α or siNC upon normoxia or hypoxia condition. The regulation of HIF-1α on NGF expression was detected by Western blot and qRT-PCR assays. All data were revealed as means ± standard deviation (SD) for no less than three independent experiments. Significant *P* values showed as ***P < 0.001. n.s means the difference was not significant

To explore how miR-21-5p enhanced NGF expression in Schwann cells, we screened the potential target genes in the TargetScan database. We found that miR-21-5p might bind to the 3′-UTR of the von Hippel-Lindau tumor-suppressor protein (VHL) mRNA (Fig. 4A). To confirm that VHL was a target of miR-21-5p, sNF96.2 cells were transfected with dual luciferase reporter plasmid containing wild type VHL 3’UTR (WT) or mutated type (MUT), which followed by transfection with miR-21-5p mimic (miR-mimic) or negative control (mimic-NC). The results showed that the luciferase activity in sNF96.2 cells was reduced upon the overexpression of miR-21-5p in the WT group, but not in the MUT group (Fig. 4B). (Fig. 4B). Meanwhile, the expression of VHL was significantly decreased or increased upon miR-21-5p overexpression or inhibition in sNF96.2 cells both at the mRNA and protein levels, respectively (Fig. 4C). VHL is a component of an E3 ubiquitin ligase complex that binds to HIF-1α, leading to its rapid degradation [23]. Coincidently, the expression of HIF-1α was significantly increased or decreased upon miR-21-5p overexpression or inhibition in sNF96.2 cells (Fig. 4C). Furthermore, the knockdown of VHL successfully rescued the expression of HIF-1α and NGF in sNF96.2 cells which was inhibited by inhibitor of miR-21-5p (Fig. 4D). On the contrary, overexpression of VHL reversed the expression of HIF-1α and NGF in sNF96.2 cells which was induced by miR-21-5p overexpression (Fig. S3A). Given that HIF-1α is a transcription factor, the data from the JASPAR database indicated that there was one potential binding site in the promoter of NGF for HIF-1α (Fig. 4E). The ChIP assay showed that hypoxia enhanced the accumulation of HIF-1α in the promoter of NGF, which was reversed by the knockdown of HIF-1α (Fig. 4F). Meanwhile, luciferase reporter assay showed that the knockdown of HIF-1α reversed the hypoxia-induced induction of NGF promoter activity in the WT group, but not in the MUT group (Fig. 4G). Moreover, the expression of NGF was significantly increased in hypoxia both at mRNA and protein levels, which was reversed by the knockdown of HIF-1α (Fig. 4H). Also, the overexpression of HIF-1α rescued the decrease in NGF expression upon VHL overexpression (Fig. S3B). Taken together, our data demonstrated that miR-21-5p enhanced NGF expression in Schwann cells by suppressing VHL/HIF-1α expression.

### NGF secreted by Schwann cells promotes the proliferation, migration, invasion, and EMT of colon cancer cells

**Fig. 5:**
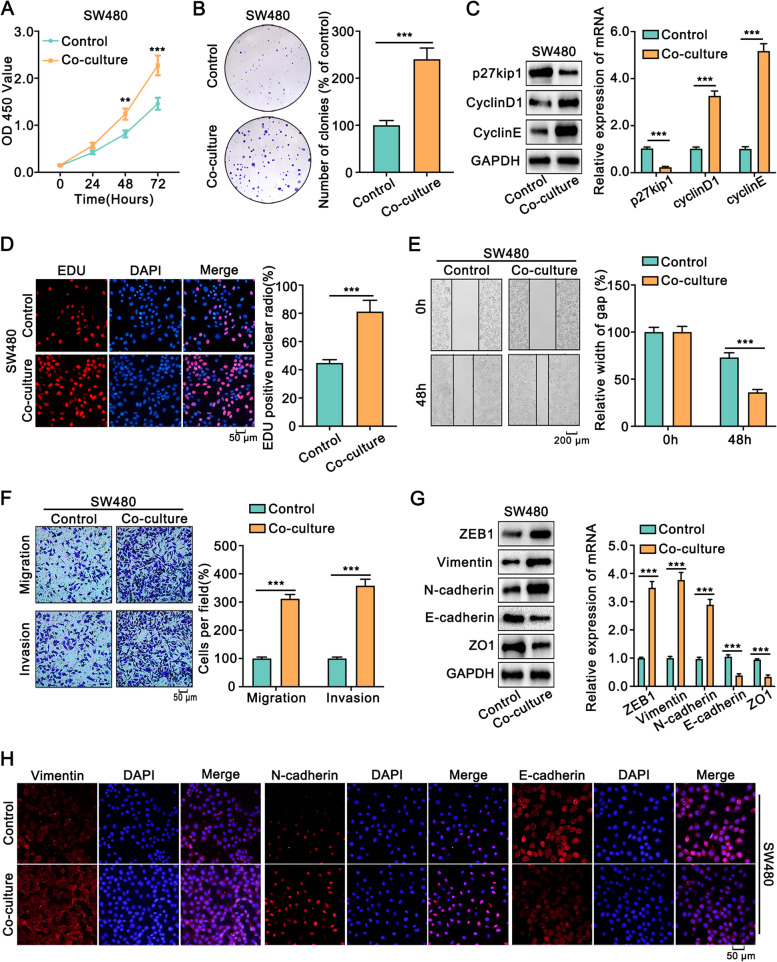
Schwann cells promoted the proliferation and metastasis of SW480 cells.** A.** The proliferation of SW480 cells co-cultured with Schwann cells or not was assessed via CCK8 for 3 days. **B.** The proliferation of SW480 cells co-cultured with Schwann cells or not was assessed via colony formation assay for 8 days. **C.** The expression of p27kip1, CyclinD1, and CyclinE of SW480 cells co-cultured with Schwann cells or not was detected by Western blot and qRT-PCR. **D.** EDU assay showed that co-cultured with Schwann cells increased the proportion of EDU-positive cells in SW480 cells. Magnification, × 200. **E.** The wound healing assays showed that co-cultured with Schwann cells significantly augmented the migrative ability of SW480 cells. **F.** The transwell and tumor invasion assays indicated that co-cultured with Schwann cells increased the migrative and invasive abilities of SW480 cells. **G.** The Western blot and qRT-PCR assays showed that co-cultured with Schwann cells increased the expression of mesenchymal markers (ZEB1, Vimentin, and N-cadherin), and decreased the expression of epithelial markers, such as E-cadherin and ZO1. **H.** Immunofluorescence assay indicated that co-cultured with Schwann cells increased mesenchymal markers of SW480cells but reduced epithelial markers. All data were revealed as means ± standard deviation (SD) for no less than three independent experiments. Significant *P* values showed as ***P < 0.001

Next, we assessed the contribution of Schwann cells in the progression of colon cancer. The CCK8 assay showed that Schwann cells significantly strengthened the viability of SW480 and HCT116 cells (Fig. 5A, S4A). Furthermore, colony formation assays demonstrated that Schwann cells increased the number and size of the colon cancer cells colonies (Fig. 5B, S4B). Moreover, Schwann cells augmented the expression of CyclinD1 and CyclinE, while attenuating the expression of p27kip1 in colon cancer cells (Fig. 5C, S4C). Also, EDU assay revealed that the proportion of EdU-positive colon cancer cells was significantly increased when co-cultured with Schwann cells (Fig. 5D, S4D).

Wound healing assays illustrated that co-culture with Schwann cells enhanced the migration of colon cancer cells (Fig. 5E, S4E). Furthermore, transwell and tumor invasion assays showed that co-culture with Schwann cells increased the migration and invasion of colon cancer cells (Fig. 5F, S4F). Given that EMT plays a significant role in cancer progression from initiation to metastasis [12], we detected the markers of EMT in colon cancer cells when co-cultured with Schwann cells. Co-culture with Schwann cells increased the expression of mesenchymal markers, such as ZEB1, Vimentin, and N-cadherin, and attenuated the expression of epithelial markers, such as E-cadherin and ZO1 in colon cancer cells (Fig. 5G, S4G). Moreover, immunohistochemical staining also showed similar results (Fig. 5H, S4H). These data demonstrated that Schwann cells promoted the migration, invasion, and EMT of colon cancer cells.

**Fig. 6:**
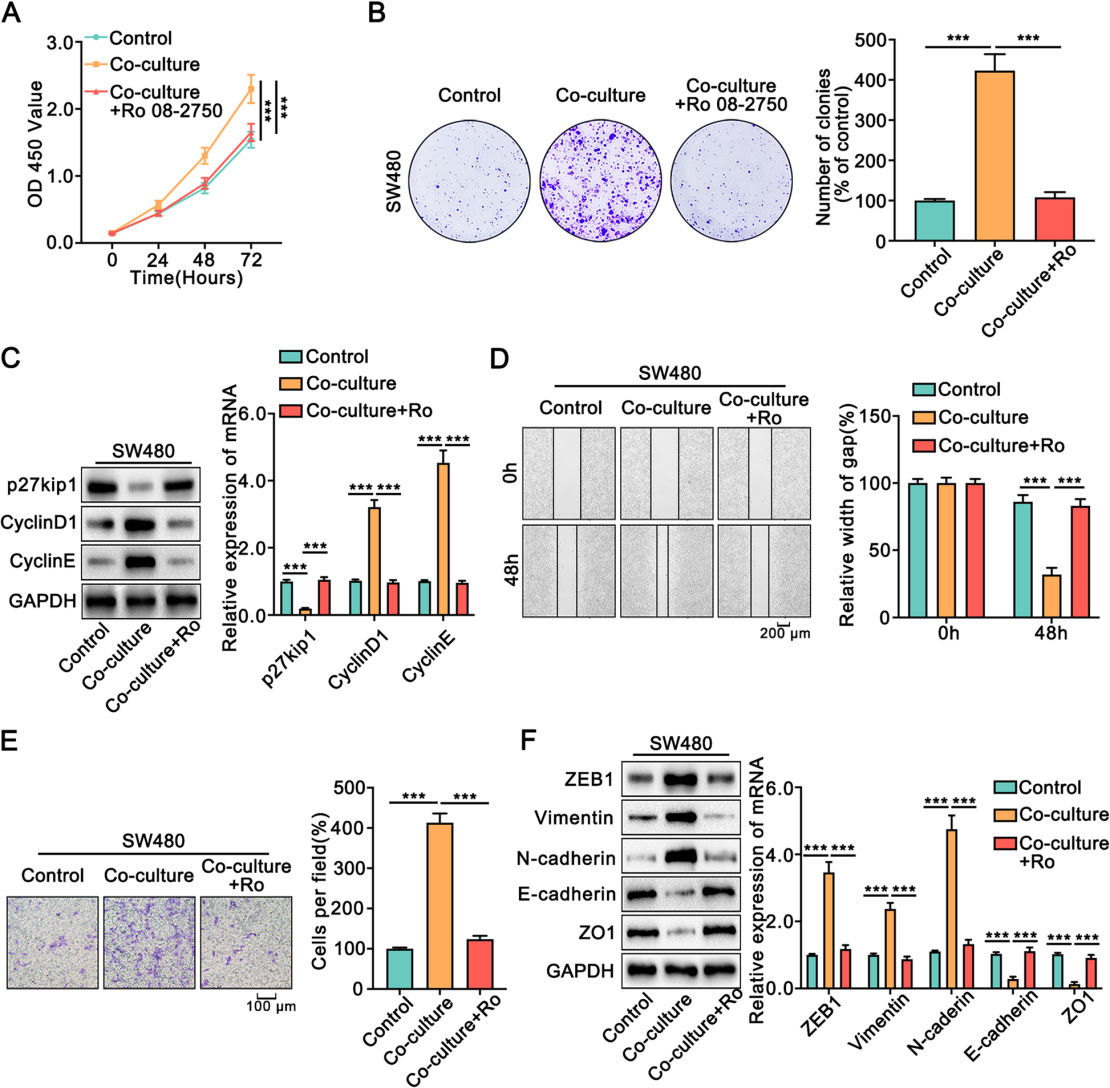
Schwann cells facilitated the proliferation, migration, invasion, and EMT of colon cancer cells through NGF.** A.** CCK8 assay showed that inhibition of NGF reversed the enhanced proliferation of SW480 cells co-cultured with Schwann cells. **B.** Colony formation assay indicated that inhibition of NGF reversed the increased number and size of the colony co-cultured with Schwann cells. **C.** Western blot and qRT-PCR showed that inhibition of NGF blocked the altered expression of p27kip1, CyclinD1, and CyclinE in SW480 cells that co-cultured with Schwann cells. **D.** The wound healing assays indicated that inhibition of NGF significantly reversed the strengthened migrative ability of SW480 cells co-cultured with Schwann cells. **E.** The transwell assays indicated that inhibition of NGF reversed the strengthened migrative ability of SW480 cells co-cultured with Schwann cells. **F.** Western blot and qRT-PCR showed that inhibition of NGF blocked the altered expression of Vimentin, N-cadherin, E-cadherin, and ZO1 in SW480 cells co-cultured with Schwann cells. Ro: Ro 08-2750, the inhibitor of NGF. All data were revealed as means ± standard deviation (SD) for no less than three independent experiments. Significant *P* values showed as ***P < 0.001. n.s means the difference was not significant

Recent studies showed that NGF accelerated the development of gastric tumorigenesis, pancreatic cancer, liver cancer, head and neck squamous cell carcinoma, prostate cancer, and chondrosarcoma [24–29]. Thus, we wondered whether Schwann cells promoted the progression of colon cancer cells through NGF. Blocking NGF significantly reversed the increase in proliferation, migration, and invasion of SW480 and HCT116 cells co-cultured with Schwann cells (Fig. 6, S5). Moreover, we added recombinant human NGF to colon cancer cells at the same concentration as that secreted by Schwann cells when co-cultured with colon cancer cells. Consistently, recombinant NGF promoted tumorigenesis (Fig. S6, S7). In conclusion, our data demonstrated that Schwann cells facilitated the proliferation, migration, invasion, and EMT of colon cancer cells by secreting NGF.

NGF promotes ZEB1 expression in colon cancer cells through secretion of NGF.

### NGF of Schwann cells accelerated ZEB1 expression of colon cancer cells by targeting TrkA/ERK/ELK1 pathway

**Fig. 7:**
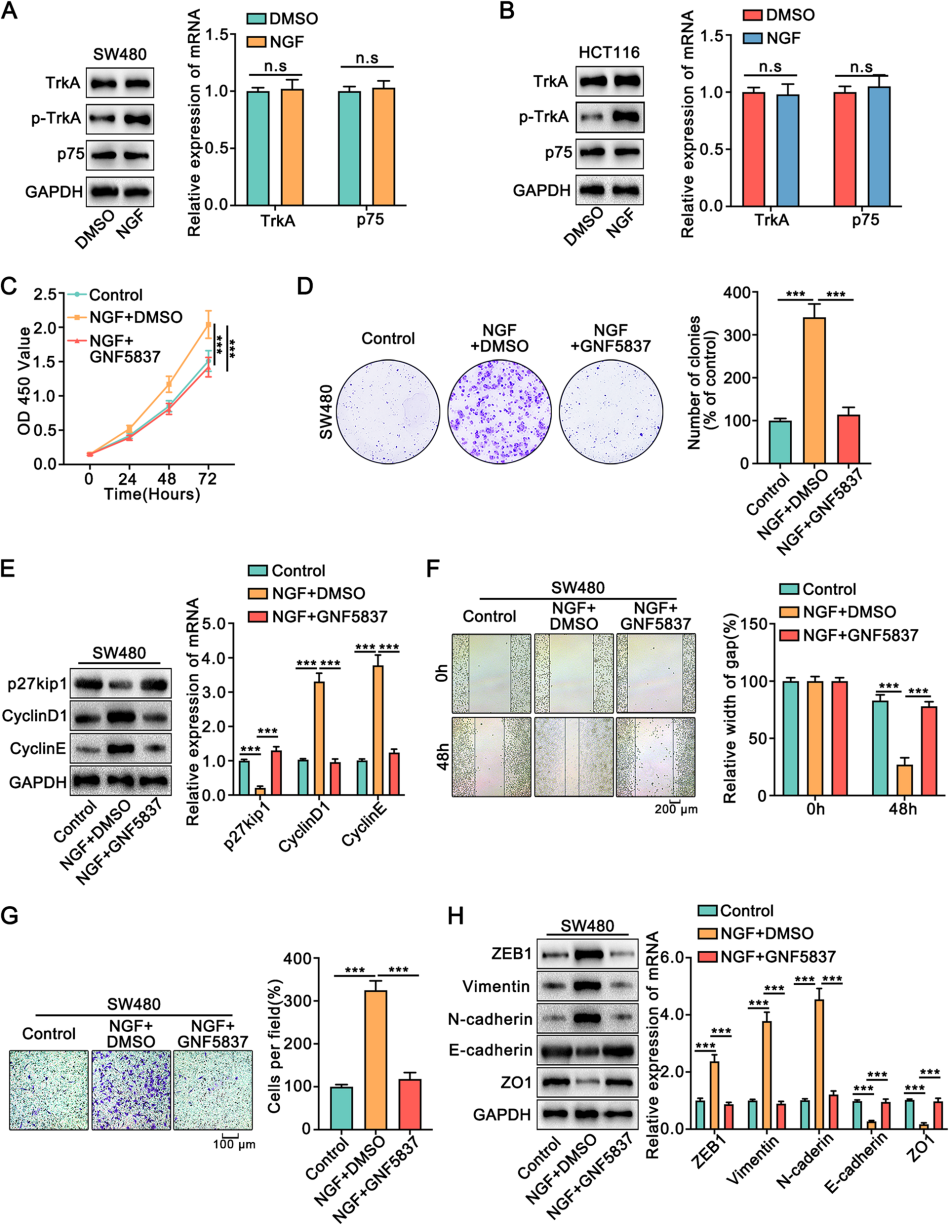
NGF modulated the proliferation and metastasis of colon cancer cells by TrkA.** A, B.** The expression of TrkA, phosphorylated TrkA, and p75 in colon cancer cells upon administration of NGF were detected by Western blot and qRT-PCR. **C.** CCK8 assay showed that inhibition of TrkA reversed the enhanced proliferation of SW480 cells upon administration of NGF. **D.** Colony formation assay indicated that inhibition of TrkA reversed the increased number and size of the colony upon administration of NGF. **E.** Western blot and qRT-PCR showed that inhibition of TrkA blocked the altered expression of p27kip1, CyclinD1, and CyclinE in SW480 cells caused by NGF. **F.** The wound healing assays indicated that inhibition of TrkA significantly reversed the strengthened migrative ability of SW480 cells upon administration of NGF. **G.** The transwell assays indicated that inhibition of TrkA reversed the strengthened migrative ability of SW480 cells upon administration of NGF. **H.** Western blot and qRT-PCR showed that inhibition of TrkA blocked the altered expression of ZEB1, Vimentin, N-cadherin, E-cadherin, and ZO1 upon administration of NGF. DMSO was used as a control. GNF5837: the inhibitor of TrkA. All data were revealed as means ± standard deviation (SD) for no less than three independent experiments. Significant *P* values showed as ***P < 0.001. n.s means the difference was not significant

NGF has two types of receptors on the cell membrane, one has a high affinity and is called TrkA, and the other has a low affinity and is called p75NTR [30]. Therefore, we detected the expression of TrkA and p75 in colon cancer cells with and without co-culture with Schwann cells. NGF increased the expression of phosphorylated TrkA in colon cancer cells, but did not affect the expression of total TrkA and p75 (Fig. 7A, B). We used GNF5837, the inhibitor of TrkA, to evaluate whether NGF promoted the progression of colon cancer through TrkA. Blocking TrkA suppressed the effect of NGF on the proliferation, migration, and invasion of colon cancer cells (Fig. 7C-H, S8). Next, we used TAT-Pep5, the inhibitor of p75, to examine whether p75 participated in NGF-induced proliferation and metastasis of colon cancer cells. TAT-Pep5 did not affect the above phenotype in colon cancer cells treated with NGF (Fig. S9, S10).

**Fig. 8:**
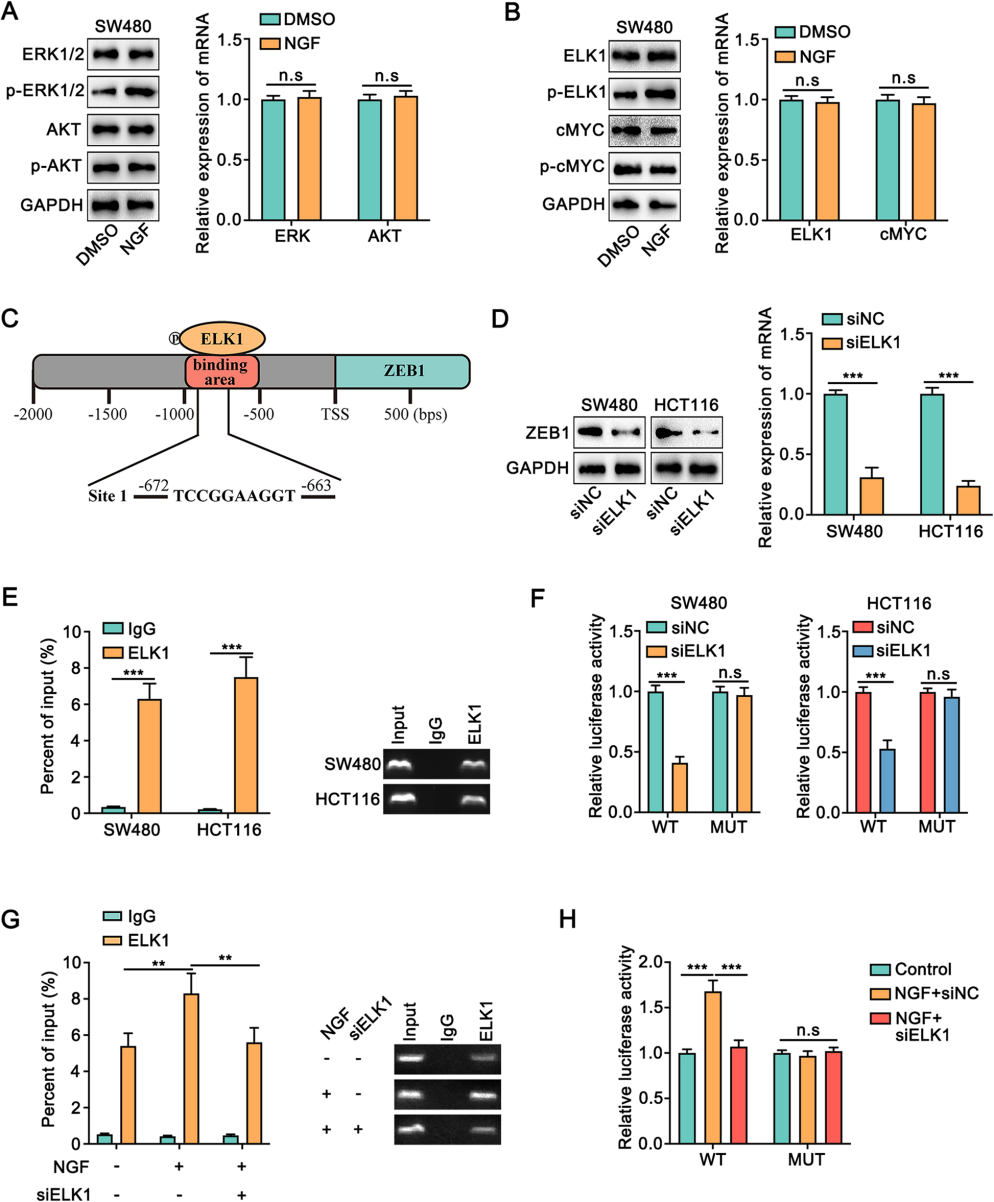
NGF facilitated the proliferation and metastasis of colon cancer through TrkA/ERK/ELK1/ZEB1** A.** The expression of ERK1/2, phosphorylated ERK1/2, AKT, and phosphorylated AKT in SW480 cells upon administration of NGF were detected by Western blot and qRT-PCR. **B.** The expression of ELK1, phosphorylated ELK1, cMYC, and phosphorylated cMYC in SW480 cells upon administration of NGF were detected by Western blot and qRT-PCR. **C.** The schematic diagram exhibited one predicted binding site between ELK1 and the promoter of ZEB1. **D.** Western blot and qRT-PCR showed that knockdown of ELK1 decreased the expression of ZEB1 in SW480 and HCT116 cells. **E.** ChIP assay demonstrated the interaction between ELK1 and promoter of ZEB1 in SW480 and HCT116 cells. **F.** The vectors containing the wild-type (WT) or mutants (MUT) of ELK1 binding sites were co-transfected with or without si-ELK1 in colon cancer cells to perform the luciferase reporter assay. WT: wild type, MUT: Site1 mutated. **G.** ChIP assay demonstrated that administration of NGF significantly strengthened the accumulation of the ZEB1 promoter region combined with anti-ELK1 antibody, while knockdown of ELK1 reversed this increase. **H.** Luciferase reported assay showed that the enhanced activity of the ZEB1 promoter upon administration of NGF conspicuously blocked when knockdown of ELK1 in the WT group, whereas there was no significant alteration in the MUT group. WT: wild type, MUT: Site1 mutated. All data were revealed as means ± standard deviation (SD) for no less than three independent experiments. Significant *P* values showed as **P < 0.01, ***P < 0.001. n.s means the difference was not significant

Previously, it was reported that the phosphorylation of TrkA led to the activation of Ras/mitogen-activated protein kinase (MAPK) signaling and phosphatidylinositol 3-kinase (PI3K)/Akt pathway [31]. Thus, we detected the expression of ERK1/2 and AKT in colon cancer cells treated with NGF. The data showed that NGF obviously increased the phosphorylation of ERK1/2, but not AKT (Fig. 8A, S11A). Furthermore, inhibition of TrkA suppressed the increase in ERK1/2 phosphorylation upon NGF administration (Fig. S11B). Moreover, LY3214996, the inhibitor of phosphorylated ERK1/2, obviously reduced the effect of NGF on the proliferation, migration, and invasion of colon cancer cells (Fig. S11C-H, S12).

The phosphorylation of ERK1/2 is known to phosphorylate and activate several transcription factors including ELK1 and cMYC [32]. Therefore, we detected the expression of ELK1 and cMYC in colon cancer cells upon treatment with NGF. The data showed that NGF enhanced the phosphorylation of ELK1, but not cMYC (Fig. 8B, S13A). Furthermore, inhibition of ERK blocked the change in ELK1 phosphorylation mediated by NGF (Fig. S13B). Moreover, knockdown of ELK1 reversed the effect of NGF on the proliferation, migration, and invasion of colon cancer cells (Fig. S13C-H, S14).

Given that ELK1 is a transcription factor, the data from the JASPAR database indicated that the presence of a potential ELK1 binding site in the promoter of ZEB1 (Fig. 8C). Furthermore, ZEB1 expression was decreased upon the knockdown of ELK1 in colon cancer cells, both at mRNA and protein levels (Fig. 8D). Meanwhile, the ChIP assay results showed that there was an obvious enrichment of the promoter of ZEB1 by the anti-ELK1 antibody (Fig. 8E). Moreover, luciferase reporter assay indicated that the activity of the ZEB1 promoter was reduced upon the knockdown of ELK1 in the WT group, but not in the MUT group (Fig. 8F). Also, the ChIP assay showed that NGF significantly increased the accumulation of anti-ELK1 antibody at the ZEB1 promoter region, while the knockdown of ELK1 reversed this (Fig. 8G). Meanwhile, luciferase reporter assay showed that the enhanced activity of the ZEB1 promoter upon NGF treatment was suppressed when ELK1 was knocked down in the WT group, whereas there was no significant alteration in the MUT group (Fig. 8H). In summary, our data demonstrated that NGF increased ZEB1 expression in colon cancer cells through TrkA/ERK/ELK1.

### Schwann cells increased miR-21-5p expression in colon cancer cells by regulating ZEB1

**Fig. 9:**
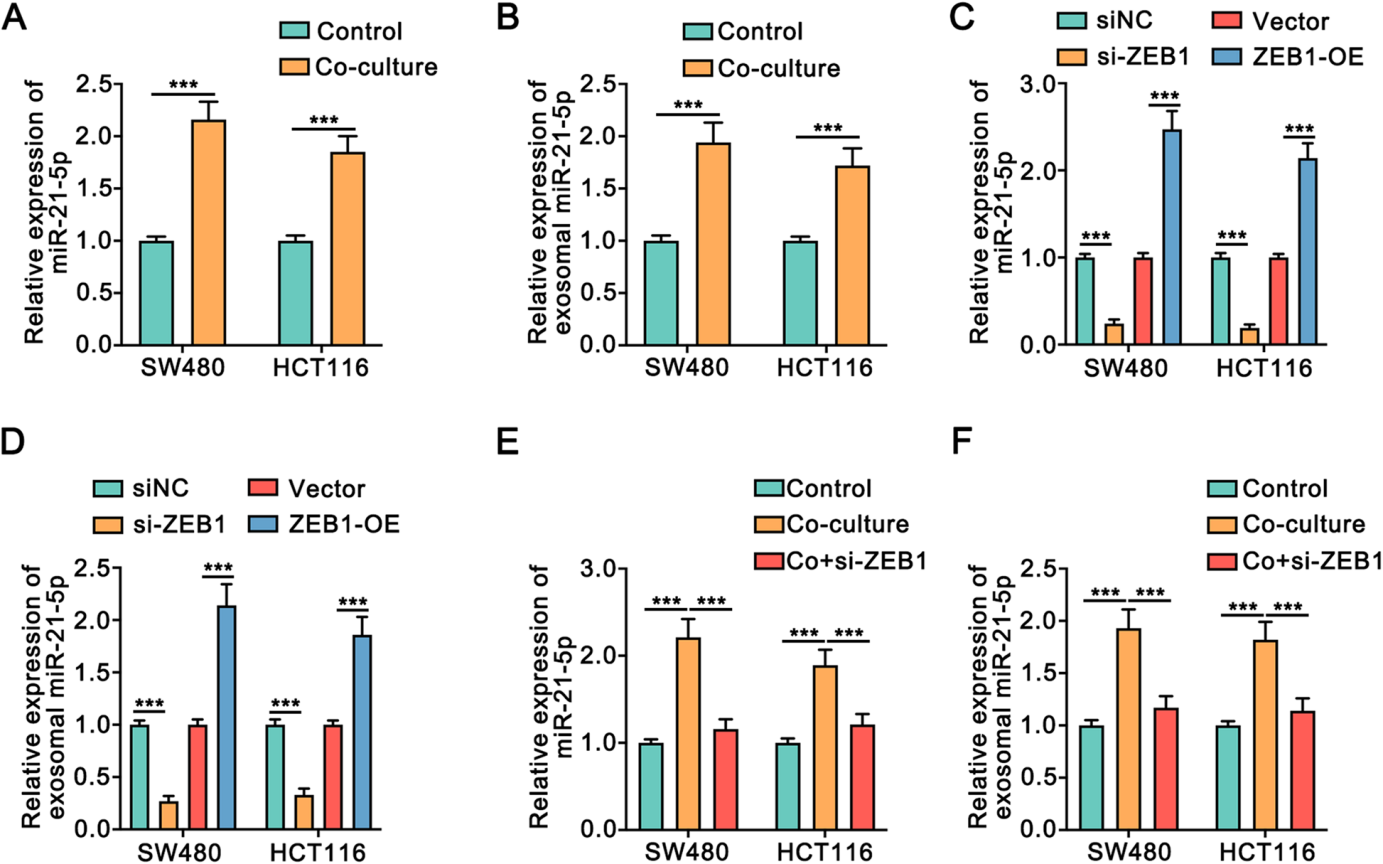
Schwann cells mutually increased miR-21-5p expression by regulating ZEB1.** A.** The qRT-PCR assays showed the expression of miR-21-5p in SW480 cells and HCT116 cells when co-cultured with Schwann cells. **B.** The qRT-PCR assays showed the expression of miR-21-5p in exosomes of SW480 cells and HCT116 cells when co-cultured with Schwann cells. **C, D.** The qRT-PCR assays showed the expression of intracellular and exosomal miR-21-5p in colon cancer cells when knockdown or overexpression of ZEB1. **E, F.** The qRT-PCR assays showed that knockdown of ZEB1 blocked the upregulated miR-21-5p expression in cells and exosomes of colon cancer when co-cultured with Schwann cells. ZEB1-OE:pcDNA3.1-ZEB1. All data were revealed as means ± standard deviation (SD) for no less than three independent experiments. Significant *P* values showed as ***P < 0.001

We further explored the potential mechanism associated with the increase in miR-21-5p expression in colon cancer. The expression of miR-21-5p was increased in SW480 cells and HCT116 cells upon co-culture with Schwann cells (Fig. 9A). Furthermore, exosomal miR-21-5p expression in SW480 cells and HCT116 cells was also elevated upon co-culture with Schwann cells (Fig. 9B). Meanwhile, Sahay et.al reported that ZEB1 promoted miR-21 expression in basal breast cancer [33]. Thus, we speculated that Schwann cells might promote miR-21-5p expression in colon cancer through ZEB1. Coincidentally, the data showed that both the intracellular and exosomal expression of miR-21-5p in colon cancer cells was significantly increased or decreased upon ZEB1 overexpression or knockdown, respectively (Fig. 9C, D). Moreover, knockdown of ZEB1 suppressed the upregulation in the intracellular and exosomal levels of miR-21-5p in colon cancer cells when co-cultured with Schwann cells (Fig. 9E, F). Thus, Schwann cells increased miR-21-5p expression in colon cancer cells by regulating ZEB1 expression.

### Schwann cells accelerated the tumorigenesis and metastasis of colon cancer in vivo

**Fig. 10:**
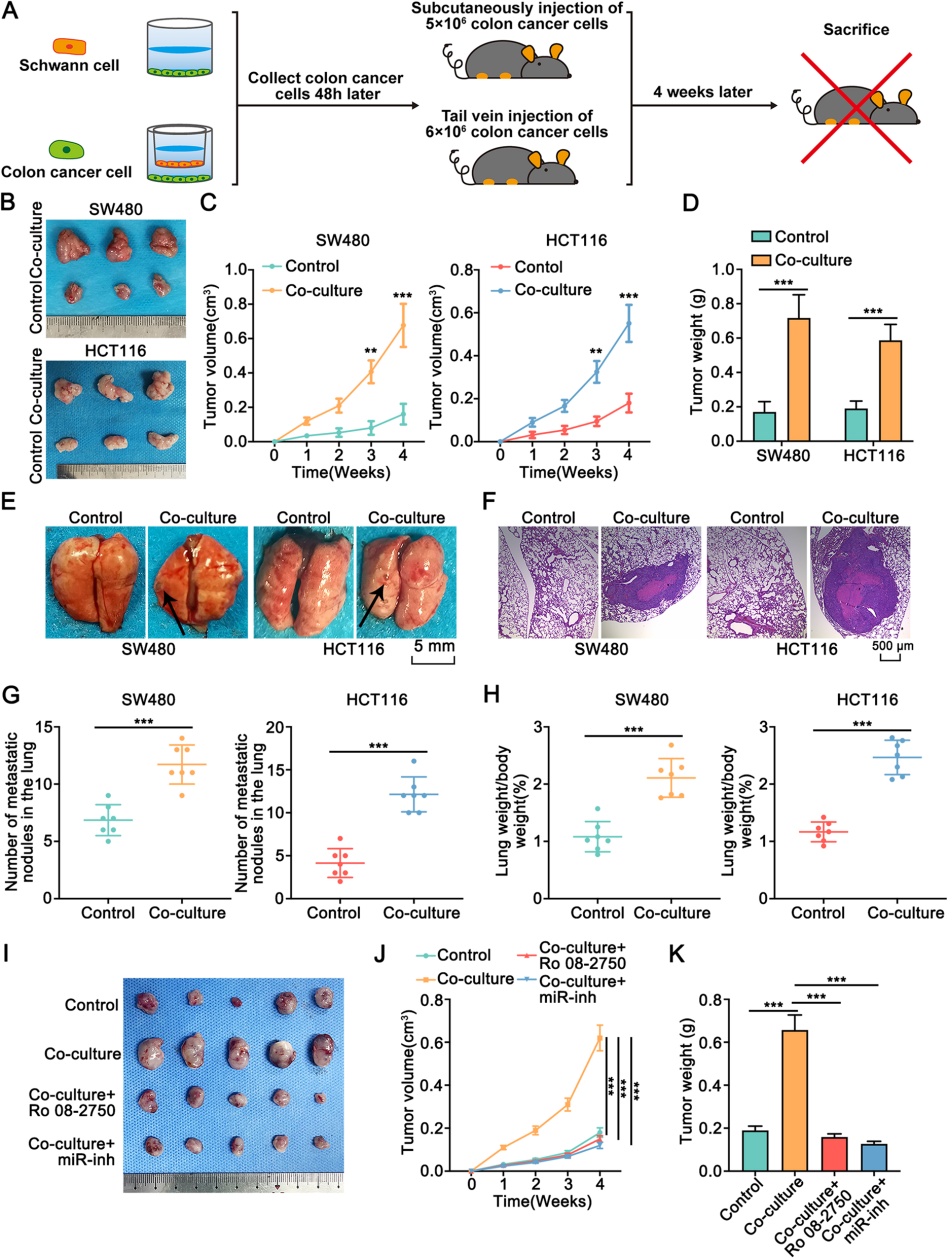
Schwann cells accelerated the tumorigenesis and metastasis of colon cancer in vivo.** A.** Schematic illustration of the animal experiment design. Colon cancer cells were cultured alone or co-cultured with Schwann cells. Forty-eight hours later, colon cancer cells were digested and injected into the subcutaneous or the tail vein of BALB/C nu/nu mice. After four weeks, the mice were sacrificed. **B-D.** Co-cultured with Schwann cells increased the volume and weight of subcutaneous tumors. **C.** The volume of transplanted tumors was measured by the Vernier caliper every 1 week. **D.** The solid tumors were peeled off and the weight was measured by electronic balance. **E.** The number of tumors in the lung was counted upon co-cultured with Schwann cell or not. **F.** HE staining showed the tumors in the lung of mice. **G-H.** The number of tumors in the lung and the lung weight/body weight (%) were significantly increased when co-cultured with Schwann cells. **I-K.** Inhibition of NGF or miR-21-5p blocked the increased volume and weight of subcutaneous tumors when co-cultured with Schwann cells. **J.** The volume of transplanted tumors was measured by the Vernier caliper every 1 week. **K.** The solid tumors were peeled off and the weight was measured by electronic balance. Ro 08-2750: the inhibitor of NGF. miR-inh: the inhibitor of miR-21-5p. All data were revealed as means ± standard deviation (SD) for no less than three independent experiments. Significant *P* values showed as **P < 0.01, and ***P < 0.001

To further explore the role of Schwann cells in the progression of colon cancer in vivo, we generated xenograft model with Balb/c nude mice (Fig. 10A). The volume and weight of subcutaneous colon cancer tumors were increased when cancer cells co-cultured with Schwann cells were injected (Fig. 10B-D). Furthermore, we injected colon cancer cells co-cultured with or without Schwann cells into the tail veins of nude mice to investigate the contribution of Schwann cells in the metastasis of colon cancer (Fig. 10A). The number of tumor nodules in the lung and the lung weight/body weight (%) were increased when cancer cells co-cultured with Schwann cells were injected (Fig. 10E-H). Moreover, we detected the expression of MIB1 (Ki67), Vimentin, N-cadherin, and E-cadherin in the subcutaneous tumor tissues by IHC. The results indicated that co-culture with Schwann cells increased the expression of MIB1 (Ki 67), Vimentin, and N-cadherin and reduced E-cadherin expression (Fig. S15). Also, inhibition of NGF or miR-21-5p significantly rescued the effect of Schwann cell co-culture on the volume and weight of subcutaneous colon tumors (Fig. 10I-K). Overall, Schwann cells accelerated the tumorigenesis and metastasis of colon cancer in vivo.

### The correlation between NGF, TrkA, ERK, ELK1, ZEB1, and miR-21-5p in human colon cancer tissues

**Fig. 11:**
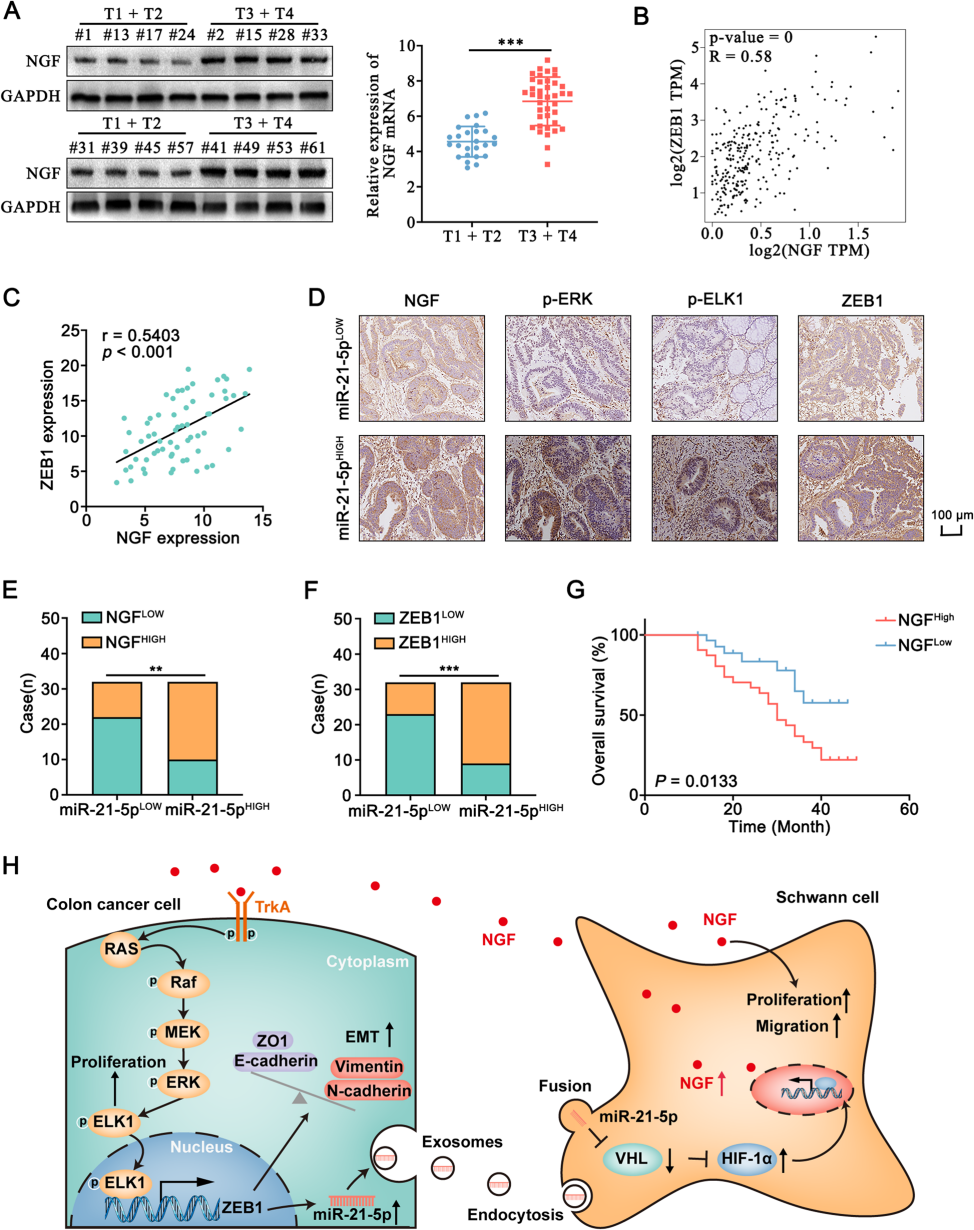
The associated expression of NGF/TrkA/ERK/ELK1/ZEB1/miR-21-5p signaling in colon cancer tissues** A.** Western blot and qRT-PCR assays showed that the NGF expression in colon cancer tissues was higher in patients with T3 and T4 than that in patients with T1 and T2. T: T staging. **B.** The relationship between NGF and ZEB1 in colon cancer tissues in the TCGA cohort from GEPIA database. **C.** The relationship between NGF and ZEB1 in colon cancer tissues was analyzed by Pearson’s correlation. **D.** The expression level of NGF, p-ERK, p-ELK1, and ZEB1 was detected by IHC in the “miR-21-5p^LOW^” or “miR-21-5p^HIGH^” group. **E, F.** The *chi*-square test identified a positive correlation between miR-21-5p and NGF and ZEB1 in the colon cancer specimens. **G.** Kaplan-Meier survival analysis indicated that the colon cancer patients in the NGF-HIGH group had a shorter overall survival time. **H.** The schematic diagram demonstrated a reciprocal feedback between Schwann cells and colon cancer cells, thereby facilitating the proliferation and metastasis of colon cancer. All data were revealed as means ± standard deviation (SD) for no less than three independent experiments. Significant *P* values showed as ***P < 0.001. n.s means the difference was not significant

Both the mRNA and protein expression levels of NGF was found to be higher in colon cancer tissues from patients with T3 and T4 stage than in patients with T1 and T2 stage (Fig. 11A). The correlation in the expression between NGF and ZEB1 was determined by Pearson’s correlation, and ZEB1 was found to be positively correlated with NGF in colon cancer tissues from our cohort and the TCGA cohort (Fig. 11B, C). Moreover, NGF expression had a positive correlation with ZEB1 expression in various other tumors (Fig. S16A, B). IHC assay indicated that the expression of NGF, p-ERK, p-ELK1, and ZEB1 in the miR-21-5p-HIGH group was higher than that in the miR-21-5p-LOW group (Fig. 11D). The *chi*-square test identified that miR-21-5p was positively correlated with NGF, p-ERK, p-ELK1, and ZEB1 in the colon cancer specimens (Fig. 11E, F, S16C, D). Moreover, Kaplan-Meier survival analysis indicated that colon cancer patients in the NGF-HIGH group had a shorter overall survival time than that in the NGF-LOW group in our cohort and TCGA cohort (Fig. 11G, S16E). Kaplan-Meier survival analysis indicated that colon cancer patients in the miR-21-5p-HIGH group had a shorter overall survival time than that in the miR-21-5p-LOW group (Fig. S16F), which was consist with previous studies [34–36]. Taken together, our data indicated that a reciprocal feedback between colon cancer cells and Schwann cells promoted the proliferation and metastasis of colon cancer (Fig. 11H).

## Discussion

Among the TME components, the peripheral nervous system (PNS) has a prominent influence on the development of CRC through perineural invasion and neoneurogenesis [37]. Schwann cells are the major glial cells in the PNS that play a major role in tumorigenesis [16]. For example, Sroka et.al reported that Schwann cells in the TME promoted the invasion of prostate and pancreatic cancer on laminin through integrin [38]. Zhou et.al indicated that Schwann cells activated the PI3K/AKT/GSK-3β pathway and augmented the expression of Snail and Twist in lung cancer cells through CXCL5/CXCR2 axis, thus promoting the EMT and metastasis of lung cancer [5]. In our study, the data demonstrated that Schwann cells were enriched in the TME of colon cancer and were associated with metastasis and poor prognosis of colon cancer.

Schwann cells migrated to colon cancer cells, rather than normal colon cells, before the onset of invasion of tumors by peripheral nerves [10]. Our data indicated that colon cancer cells facilitated the proliferation and migration of Schwann cells. Schwann cells regulated the expression of various neurotrophic factors including NGF [16]. NGF consists of two intertwined 13-kDa beta chains joined by disulfide bonds in a typical cysteine knot, usually a dimer [39]. NGF is known to promote the proliferation and migration of Schwann cells [17, 18]. In our study, we found that colon cancer cells promoted the proliferation and migration of Schwann cells by stimulating the secretion of NGF from Schwann cells.

Exosomes are a major component of extracellular vesicles (EVs) and their size ranges between 30 to 150 nm [21]. Recent studies revealed that exosomes remodeled the TME and facilitated the metastasis of several types of tumors by delivering RNA, DNA, or proteins [40, 41]. MicroRNAs (miRNAs) are 17-24 nt long small noncoding RNAs, are highly abundant in the exosomes, playing an important role in intercellular communication [21, 42]. Zhao et.al reported that miR-934 was packaged in exosomes and induced M2 macrophage polarization by activating the PI3K/AKT signaling pathway, thereby promoting CRC liver metastasis [43]. Hu et.al showed that exosomal miR-92a-3p secreted by cancer-associated fibroblasts facilitated the stemness, EMT, metastasis, and chemotherapy resistance of CRC cells through Wnt/β-catenin axis [44]. However, the function of exosomes in the communication between Schwann cells and colon cancer cells is still unclear. Our data showed that colon cancer cells augmented the expression of NGF in Schwann cells through exosomes, which in turn facilitated the progression of colon cancer.

Accumulating evidence has shown the role of miR-21-5p in the progression and dissemination of CRC [36, 45]. For example, Yu et.al demonstrated that miR-21-5p facilitated the proliferation and invasion of colon adenocarcinoma through CHL1 [45]. He et.al reported that exosomal miR-21-5p was delivered from CRC cells to endothelial cells, thereby promoting angiogenesis and vascular permeability through the activation of the β-catenin pathway [46]. In our study, we found that exosomal miR-21-5p derived from colon cancer cells promoted the expression of NGF in Schwann cells. Furthermore, inhibition of miR-21-5p significantly suppressed the Schwann cell induced progression of colon cancer.

miRNAs typically exert their functions by binding to the 3’UTR of their target genes [47]. Several studies demonstrated that miR-21 directly decreased the expression of VHL in glioblastomas, papillary thyroid carcinoma, and pancreatic cancer [48–50]. Moreover, Cai et.al reported that miR-21-5p augmented the proliferation, migration, and invasion and inhibited apoptosis of cervical cancer cells through the downregulation of VHL [51]. In the current study, gain- and loss-of-function experiments revealed that miR-21-5p downregulated VHL expression in Schwann cells. VHL is a component of an E3 ubiquitin ligase complex that binds to HIF-1α, leading to its rapid degradation [23]. Our data indicated that miR-21-5p increased HIF-1α expression in Schwann cells by downregulating VHL expression. Furthermore, ChIP and luciferase assays demonstrated that HIF-1α bound to the promoter region of NGF and facilitated its transcription in Schwann cells.

NGF not only has a prominent impact on the nervous system, but also plays a major role in the growth, invasion, and metastasis of several types of solid tumors [52–55]. Hayakawa et.al found that the overexpression of NGF significantly accelerated the growth and invasion of gastric tumors [27]. Lei et.al revealed that NGF contributed to the proliferation and metastasis of pancreatic tumors [54]. Moreover, it was reported that NGF promoted EMT in several tumors [53, 55]. Our data showed that NGF facilitated the proliferation, migration, invasion, and EMT of colon cancer cells, supporting the function of NGF in solid tumors. Furthermore, blocking NGF suppressed the progression of Schwann cell-induced progression of colon cancer.

It was reported that NGF regulated the function of several types of tumor cells through TrkA [56–58]. When NGF binds to TrkA, TrkA automatically phosphorylates and activates the downstream second-messenger cascade, mediating important biological effects on the survival, proliferation, and migration of cells [59, 60]. Rayego-Mateos et.al showed that TrkA regulated the EMT process in renal cells [61]. In our study, NGF significantly increased the expression of phosphorylated TrkA in colon cancer cells. Inhibition of TrkA, but not p75, reversed the effect of NGF on the proliferation, migration, and invasion of colon cancer cells. It was reported that NGF activated the MAPK signaling pathway through TrkA in several tumors [62, 63]. Descamps et.al revealed that NGF promoted the proliferation and survival of human breast cancer cells through the activation of TrkA/MAPK axis [62]. Okada et.al showed that NGF promoted MMP-2 expression and accelerated the invasion of human pancreatic cancer by activating the MAPK pathway [63]. In our study, we found that NGF obviously increased the phosphorylation of ERK and ELK1 in colon cancer cells through TrkA. Furthermore, inhibition of ERK phosphorylation or knockdown of ELK1 reversed the effect of NGF on the proliferation, migration, and invasion of colon cancer cells. ZEB1 is a transcription factor characterized by N-terminal and C-terminal CYS2-HIS2 zinc fingers separated by a homeobox domain [64]. ZEB1 is known to induce EMT by suppressing the expression of E-cadherin [65]. Chiu et.al reported that ERK promoted the expression of ZEB1 in pemetrexed resistant lung cancer cells [66]. Coincidentally, our data indicated that ELK1 bound to ZEB1 promoter ZEB1 and facilitated its transcription. Moreover, Schwann cells boosted the expression of miR-21-5p in colon cancer cells by upregulating ZEB1.

## Conclusions

In summary, our study identified a reciprocal feedback loop between Schwann cells and colon cancer cells, which facilitated the proliferation and metastasis of colon cancer cells. Targeting NGF and exosomal miR-21-5p may be a potential therapeutic strategy for treating colon cancer. However, further studies are required to unravel the precise molecular mechanisms underlying the cross-talk between Schwann cells and colon cancer cells.

## Supplementary Information

**Additional file 1 Fig. S1. **Colon cancer cells promoted the proliferation and migration of Schwann cells by stimulating their secretion of NGF. **Fig. S2. **Exosomes derived from colon cancer cells facilitated the expression of NGF in Schwann cells via miR-21-5p. **Fig. S3 **miR-21-5p promoted the expression of NGF in Schwann cells through VHL/HIF-1α. **Fig. S4. **Schwann cells promoted the proliferation and metastasis of HCT116 cells. **Fig. S5. **Schwann cells facilitated the proliferation, migration, invasion, and EMT of colon cancer cells through NGF. **Fig. S6. **NGF facilitated the proliferation, migration, invasion, and EMT of SW480 cells. **Fig. S7. **NGF facilitated the proliferation, migration, invasion, and EMT of HCT116 cells. **Fig. S8. **NGF modulated the proliferation and metastasis of colon cancer cells by TrkA. **Fig. S9. **P75 did not involve in the NGF-induced proliferation and metastasis of colon cancer cells. **Fig. S10. **P75 did not involve in the NGF-induced proliferation and metastasis of colon cancer cells. **Fig. S11. **NGF modulated the proliferation and metastasis of colon cancer cells through ERK. **Fig. S12. **NGF modulated the proliferation and metastasis of colon cancer cells through ERK. **Fig. S13. **NGF modulated the proliferation and metastasis of colon cancer cells via ERK/ELK1. **Fig. S14. **NGF modulated the proliferation and metastasis of colon cancer cells via ERK/ELK1. **Fig. S15. **Schwann cells accelerated the tumorigenesis and metastasis of colon cancer in vivo. **Fig. S16. **The associated expression of NGF/TrkA/ERK/ELK1/ZEB1/miR-21-5p signaling in colon cancer tissues**.** **Table S1. **Clinicopathological characteristics of colon cancer patients. **Table S2. **Primers of genes in this research for qRT-PCR. **Table S3.** Details of primary antibodies applied in this study.

## Data Availability

The datasets used and/or analysed during the current study are available from the corresponding author on reasonable request.

## Abbreviations

CRC: Colorectal cancer
TME: Tumor microenvironment
PNS: Peripheral nervous system
EMT: Epithelial-mesenchymal transition
GFAP: Glial-fibrillary-acidic protein
MAPK: Ras/mitogen-activated protein kinase
PI3K: Phosphatidylinositol 3-kinase
ChIP: Chromatin immunoprecipitation
EVs: Extracellular vesicles.
VHL: Von Hippel-Lindau tumor-suppressor protein.
