# Supplementary material for: A reciprocal feedback between colon cancer cells and Schwann cells promotes the proliferation and metastasis of colon cancer
Source: J Exp Clin Cancer Res. 2022 Dec 15;41:348. doi: 10.1186/s13046-022-02556-2 (PMC9753336; doi:10.1186/s13046-022-02556-2)
Supplement: Supplementary file 1 — Additional file 1 Fig. S1. Colon cancer cells promoted the proliferation and migration of Schwann cells by stimulating their secretion of NGF. Fig. S2. Exosomes derived from colon cancer cells facilitated the expression of NGF in Schwann cells via miR-21-5p. Fig. S3 miR-21-5p promoted the expression of NGF in Schwann cells through VHL/HIF-1α. Fig. S4. Schwann cells promoted the proliferation and metastasis of HCT116 cells. Fig. S5. Schwann cells facilitated the proliferation, migration, invasion, and EMT of colon cancer cells through NGF. Fig. S6. NGF facilitated the proliferation, migration, invasion, and EMT of SW480 cells. Fig. S7. NGF facilitated the proliferation, migration, invasion, and EMT of HCT116 cells. Fig. S8. NGF modulated the proliferation and metastasis of colon cancer cells by TrkA. Fig. S9. P75 did not involve in the NGF-induced proliferation and metastasis of colon cancer cells. Fig. S10. P75 did not involve in the NGF-induced proliferation and metastasis of colon cancer cells. Fig. S11. NGF modulated the proliferation and metastasis of colon cancer cells through ERK. Fig. S12. NGF modulated the proliferation and metastasis of colon cancer cells through ERK. Fig. S13. NGF modulated the proliferation and metastasis of colon cancer cells via ERK/ELK1. Fig. S14. NGF modulated the proliferation and metastasis of colon cancer cells via ERK/ELK1. Fig. S15. Schwann cells accelerated the tumorigenesis and metastasis of colon cancer in vivo. Fig. S16. The associated expression of NGF/TrkA/ERK/ELK1/ZEB1/miR-21-5p signaling in colon cancer tissues. Table S1. Clinicopathological characteristics of colon cancer patients. Table S2. Primers of genes in this research for qRT-PCR. Table S3. Details of primary antibodies applied in this study. [file 13046_2022_2556_MOESM1_ESM.docx]

**
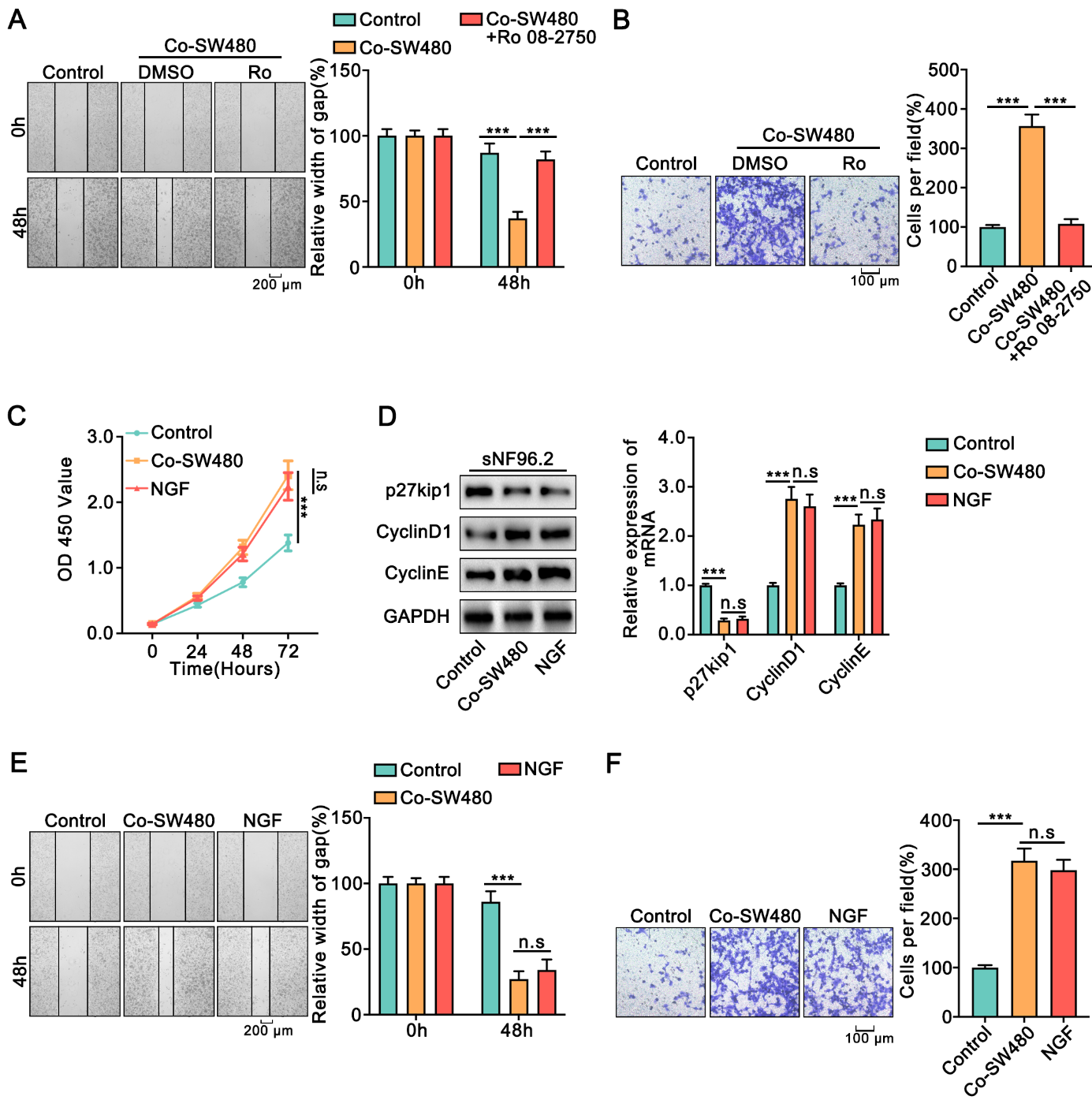
**

**Figure S1.** **Colon cancer cells promoted the proliferation and migration of Schwann cells by stimulating their secretion of NGF. (A).** The wound healing assays indicated that inhibition of NGF significantly reversed the strengthened migrative ability of Schwann cells co-cultured with SW480 cells. **(B).** The transwell assays indicated that inhibition of NGF reversed the strengthened migrative ability of Schwann cells co-cultured with SW480 cells. **(C).** CCK8 assay showed that NGF promoted the proliferation of Schwann cells. **(D).** Western blot and qRT-PCR showed that NGF augmented the expression of p27kip1, Cyclin D1, and Cyclin E in Schwann cells. **(E).** The wound healing assays indicated that NGF strengthened the migrative ability of Schwann cells. **(F).** The transwell assays indicated that NGF enhanced the migrative ability of Schwann cells. All data were revealed as means ± standard deviation (SD) for no less than three independent experiments. Significant *P* values showed as ****P <* 0.001. n.s means the difference was not significant.

**
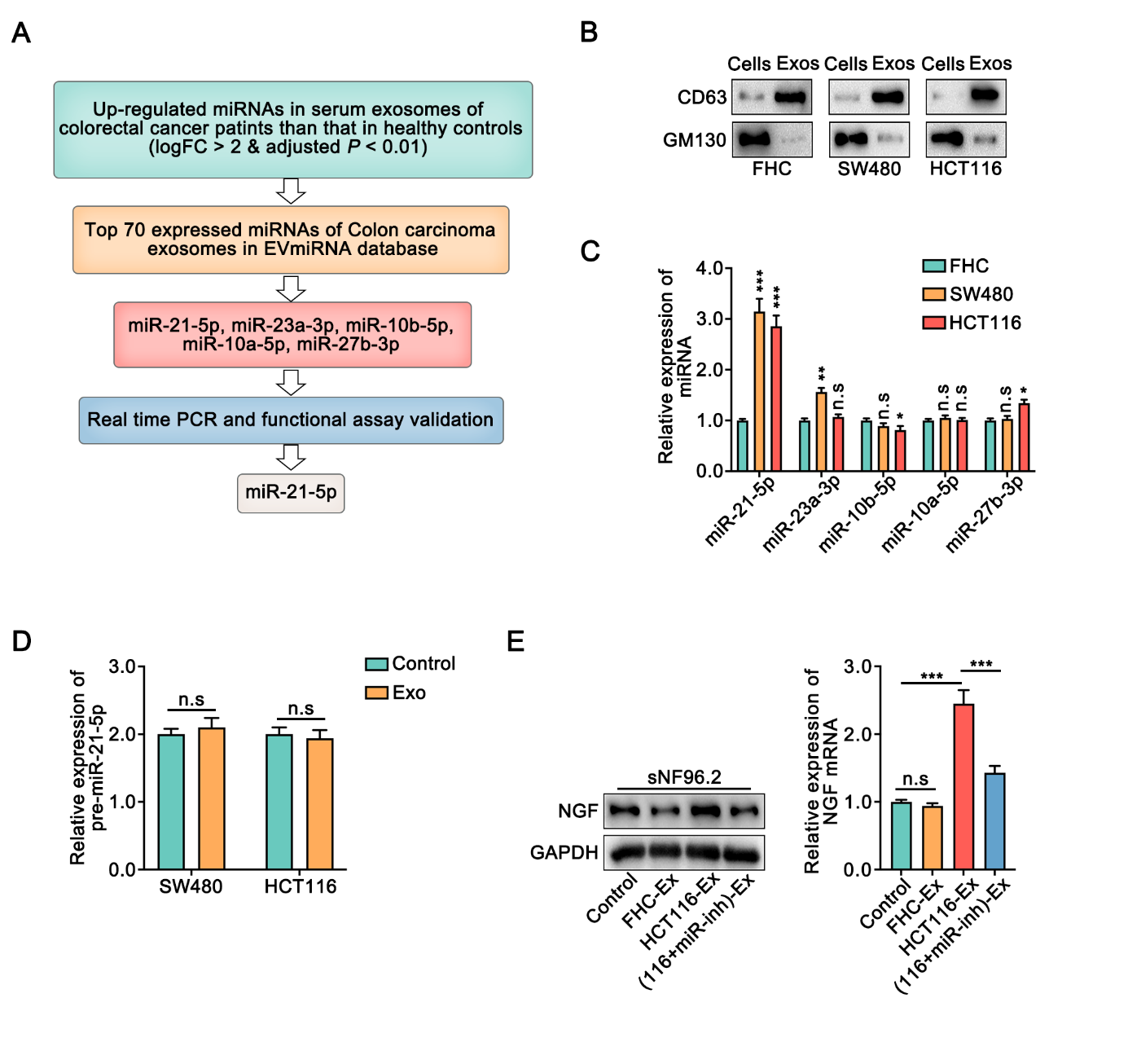
**

**Figure S2.** **Exosomes derived from colon cancer cells facilitated the expression of NGF in Schwann cells via miR-21-5p. (A).** A schematic diagram of screening high expressed miRNAs in the exosomes of CRC. **(B).** Western blot assay analyzed the exosome markers CD63 and GM130 in the protein extracted from cells and exosomes. **(C).** The qRT-PCR showed the expression of five miRNAs in the FHC, SW480, and HCT116 cells. **(D).** The qRT-PCR showed the expression of pre-miR-21-5p in Schwann cells with or without the exosomes from SW480, and HCT116 cells. **(E).** Western blot and qRT-PCR showed that inhibition of miR-21-5p in HCT116 cells significantly blocked the increased expression of NGF in Schwann cells incubated with the exosome of the HCT116 cells. All data were revealed as means ± standard deviation (SD) for no less than three independent experiments. Significant *P* values showed as ****P <* 0.001. n.s means the difference was not significant.

**
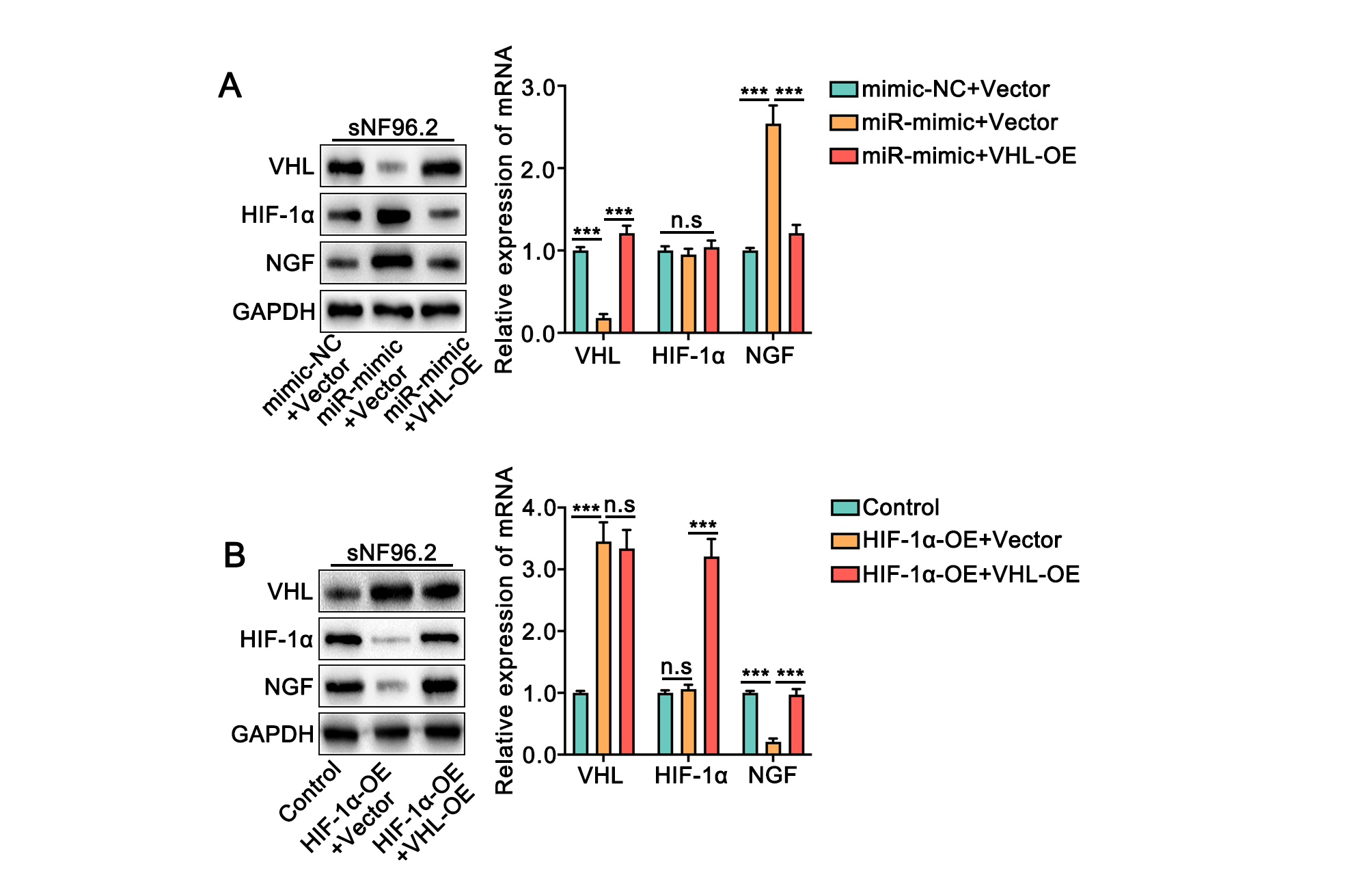
**

**Figure S3 miR-21-5p promoted the expression of NGF in Schwann cells through VHL/****HIF-1α. (A).** Effect of miR-21-5p/VHL/HIF-1α pathway on NGF expression in sNF96.2 cells. sNF96.2 cells were co-transfected with miR-21-5p mimic (miR-mimic) and VHL overexpression plasmid (VHL-OE), as well as associated negative control (miR-NC, Vector), respectively. The expression of VHL/HIF-1α/NGF was evaluated by Western blot and qRT-PCR assay. **(B).** Effect of VHL/HIF-1α pathway on NGF expression in sNF96.2 cells. sNF96.2 cells were co-transfected with HIF-1α overexpression plasmid (HIF-1α-OE) and VHL-OE, as well as empty vector plasmid (Vector). The expression of VHL/HIF-1α/NGF was evaluated by Western blot and qRT-PCR assay. All data were revealed as means ± standard deviation (SD) for no less than three independent experiments. Significant *P* values showed as ****P <* 0.001. n.s means the difference was not significant.

**
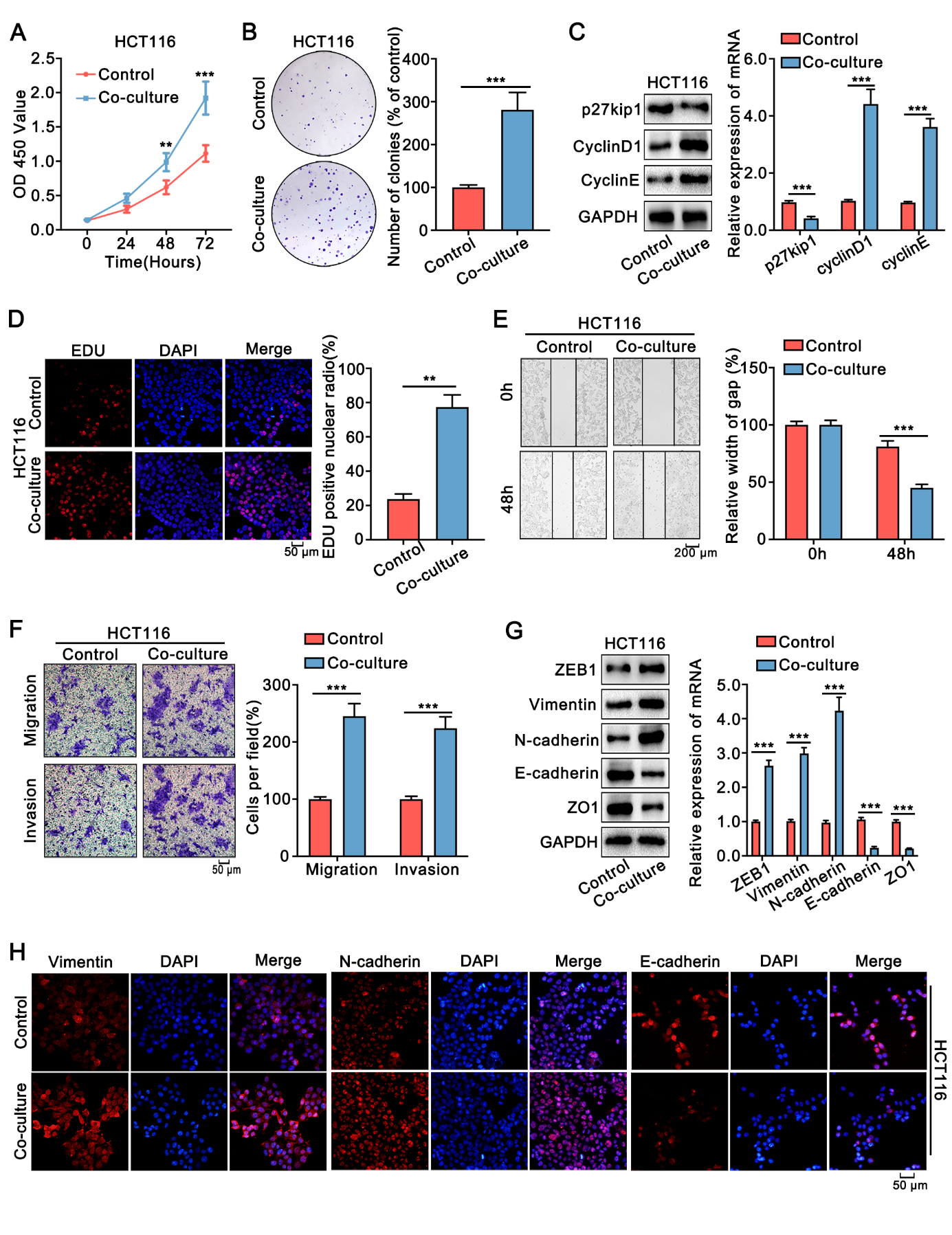
**

**Figure S4. Schwann cells promoted the proliferation and metastasis of HCT116 cells.** **(A).** The proliferation of HCT116 cells co-cultured with Schwann cells or not was assessed via CCK8 for 3 days. **(B).** The proliferation of HCT116 cells co-cultured with Schwann cells or not was assessed via colony formation assay for 8 days. **(C).** The expression of p27kip1, CyclinD1, and CyclinE of HCT116 cells co-cultured with Schwann cells or not was detected by Western blot and qRT-PCR. **(D).** EDU assay showed that co-cultured with Schwann cells increased the proportion of EDU-positive cells in HCT116 cells. Magnification, ×200. **(E).** The wound healing assays showed that co-cultured with Schwann cells significantly augmented the migrative ability of HCT116 cells. **(F).** The transwell and tumor invasion assays indicated that co-cultured with Schwann cells increased the migrative and invasive abilities of HCT116 cells. **(G).** The Western blot and qRT-PCR assays showed that co-cultured with Schwann cells increased the expression of mesenchymal markers (ZEB1, Vimentin, and N-cadherin), and decreased the expression of epithelial markers, such as E-cadherin and ZO1. **(H).** Immunofluorescence assay indicated that co-cultured with Schwann cells increased mesenchymal markers of HCT116cells but reduced epithelial markers. All data were revealed as means ± standard deviation (SD) for no less than three independent experiments. Significant *P* values showed as ****P <* 0.001.

**
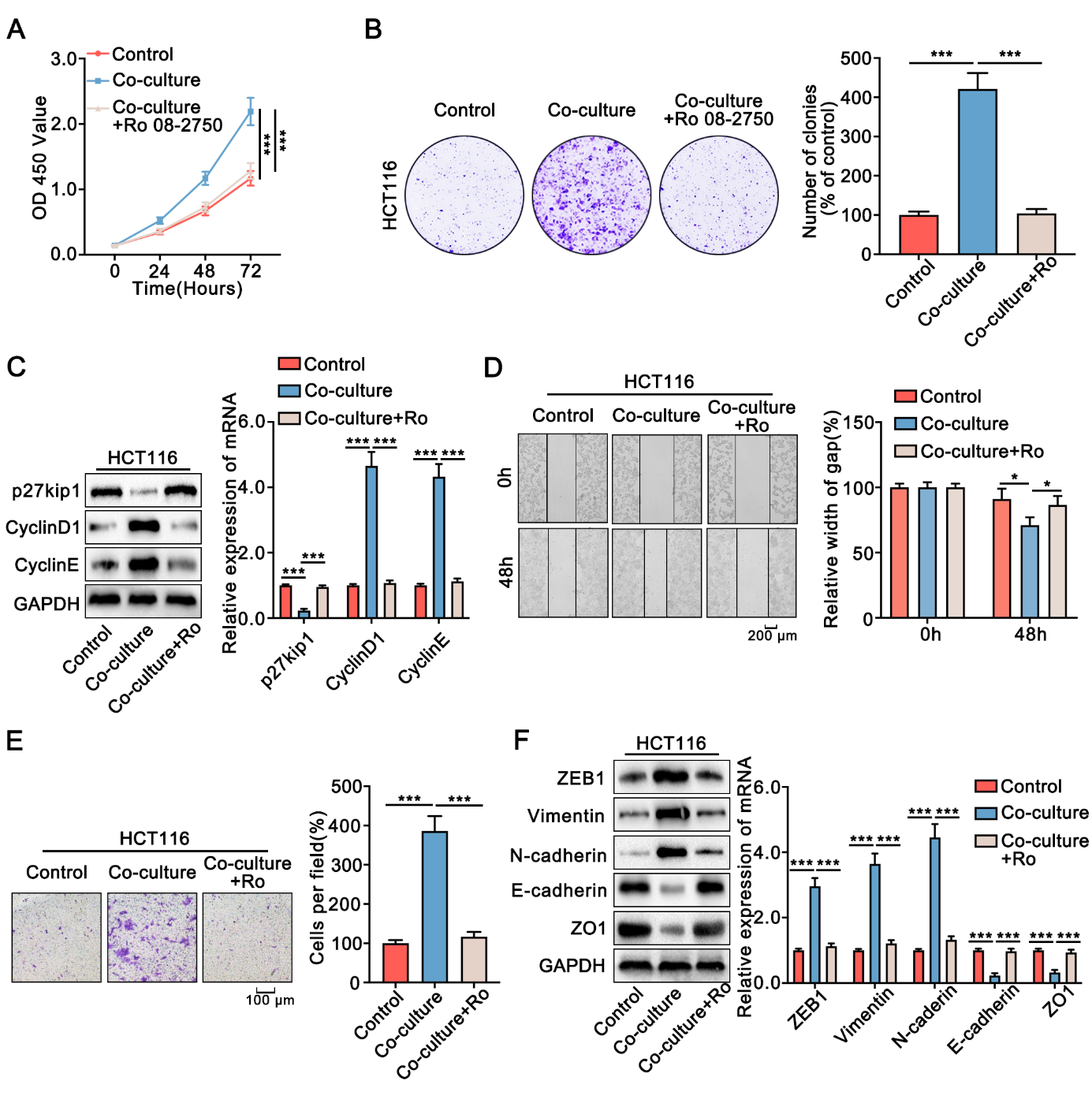
**

**Figure S5. Schwann cells facilitated** **the proliferation, migration, invasion, and EMT of colon cancer cells through NGF.** **(A).** CCK8 assay showed that inhibition of NGF reversed the enhanced proliferation of HCT116 cells co-cultured with Schwann cells. **(B).** Colony formation assay indicated that inhibition of NGF reversed the increased number and size of the colony co-cultured with Schwann cells. **(C).** Western blot and qRT-PCR showed that inhibition of NGF blocked the altered expression of p27kip1, CyclinD1, and CyclinE in HCT116 cells that co-cultured with Schwann cells. **(D).** The wound healing assays indicated that inhibition of NGF significantly reversed the strengthened migrative ability of HCT116 cells co-cultured with Schwann cells. **(E).** The transwell assays indicated that inhibition of NGF reversed the strengthened migrative ability of HCT116 cells co-cultured with Schwann cells. **(F).** Western blot and qRT-PCR showed that inhibition of NGF blocked the altered expression of Vimentin, N-cadherin, E-cadherin, and ZO1 in HCT116 cells co-cultured with Schwann cells. Ro: Ro 08-2750, the inhibitor of NGF. All data were revealed as means ± standard deviation (SD) for no less than three independent experiments. Significant *P* values showed as ****P <* 0.001. n.s means the difference was not significant.

**
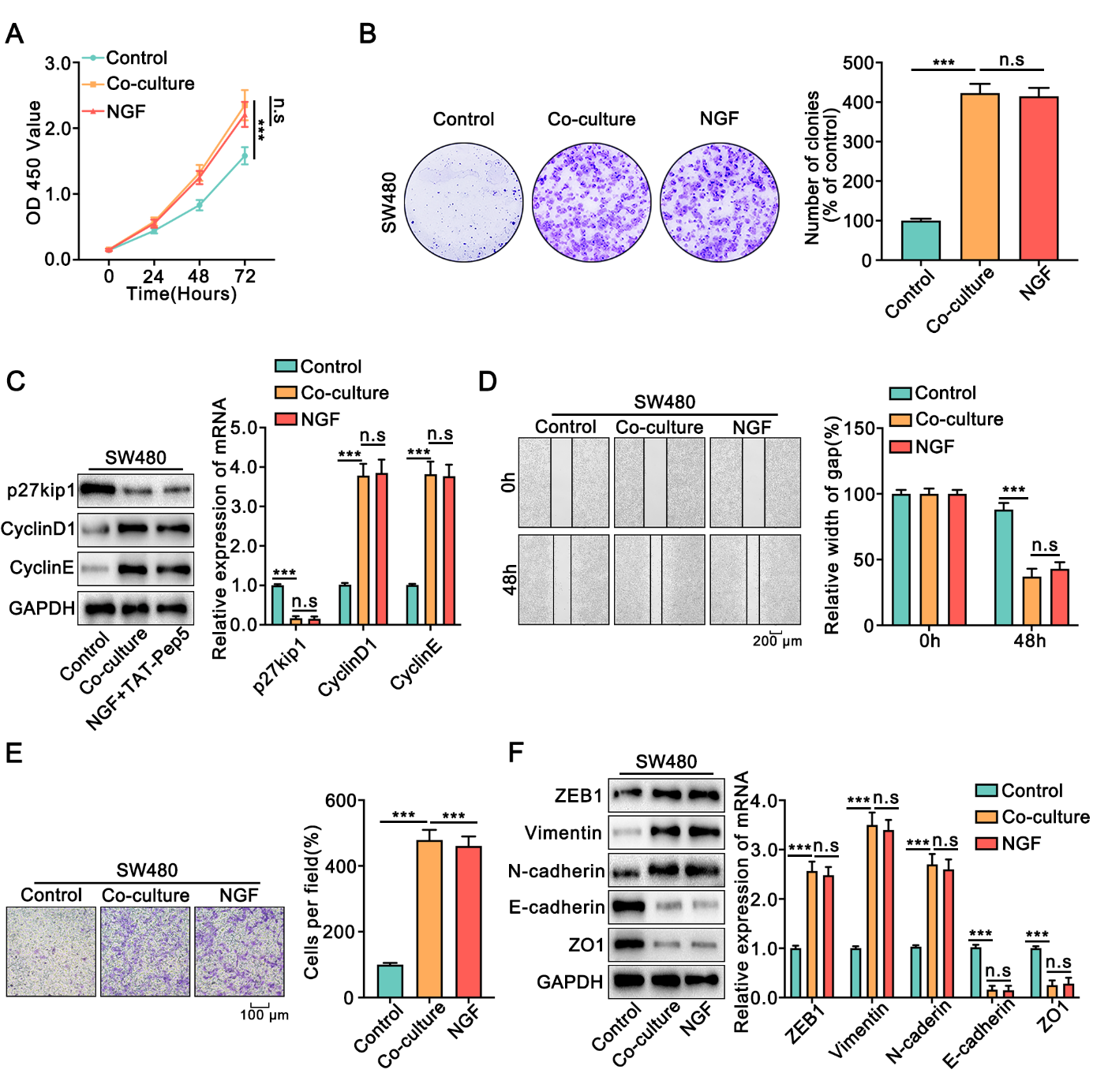
**

**Figure S6. NGF facilitated the proliferation, migration, invasion, and EMT of SW480 cells. (A).** CCK8 assay showed that NGF promoted the proliferation of SW480 cells. **(B).** The colony formation assay indicated that NGF increased the number and size of the colony. **(C).** Western blot and qRT-PCR showed that NGF augmented the expression of p27kip1, CyclinD1, and CyclinE in SW480 cells. **(D).** The wound healing assays indicated that NGF strengthened the migrative ability of SW480 cells. **(E).** The transwell assays indicated that NGF enhanced the migrative ability of SW480 cells. **(F).** Western blot and qRT-PCR showed that NGF facilitated the expression of ZEB1, Vimentin, and N-cadherin, but reduced the expression of E-cadherin, and ZO1 in SW480 cells. All data were revealed as means ± standard deviation (SD) for no less than three independent experiments. Significant *P* values showed as ****P <* 0.001. n.s means the difference was not significant.

**
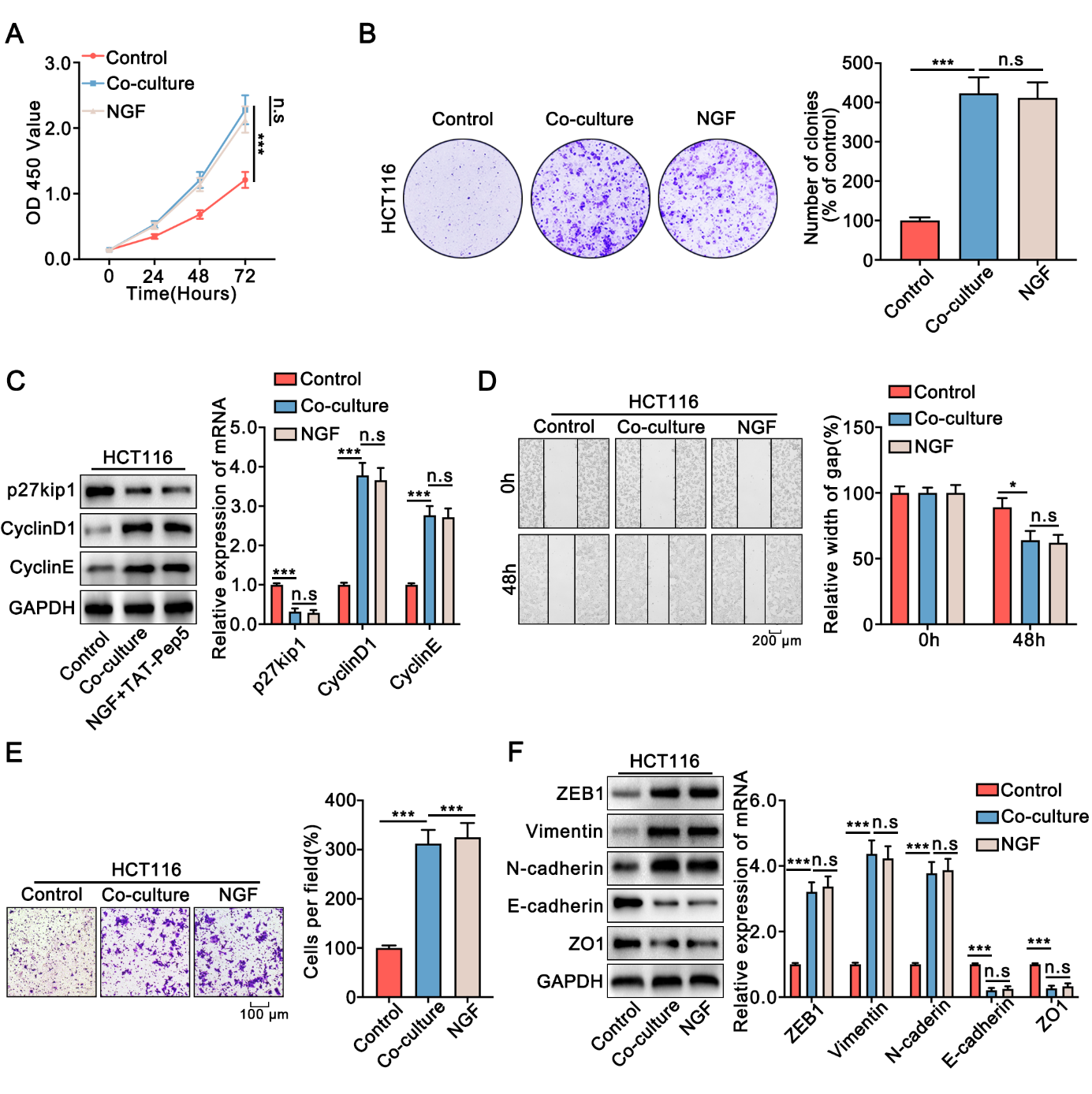
**

**Figure S7. NGF facilitated the proliferation, migration, invasion, and EMT of HCT116 cells. (A).** CCK8 assay showed that NGF promoted the proliferation of HCT116 cells. **(B).** The colony formation assay indicated that NGF increased the number and size of the colony. **(C).** Western blot and qRT-PCR showed that NGF augmented the expression of p27kip1, CyclinD1, and CyclinE in HCT116 cells. **(D).** The wound healing assays indicated that NGF strengthened the migrative ability of HCT116 cells. **(E).** The transwell assays indicated that NGF enhanced the migrative ability of HCT116 cells. **(F).** Western blot and qRT-PCR showed that NGF facilitated the expression of ZEB1, Vimentin, and N-cadherin, but reduced the expression of E-cadherin, and ZO1 in HCT116 cells. All data were revealed as means ± standard deviation (SD) for no less than three independent experiments. Significant *P* values showed as ****P <* 0.001. n.s means the difference was not significant.

**
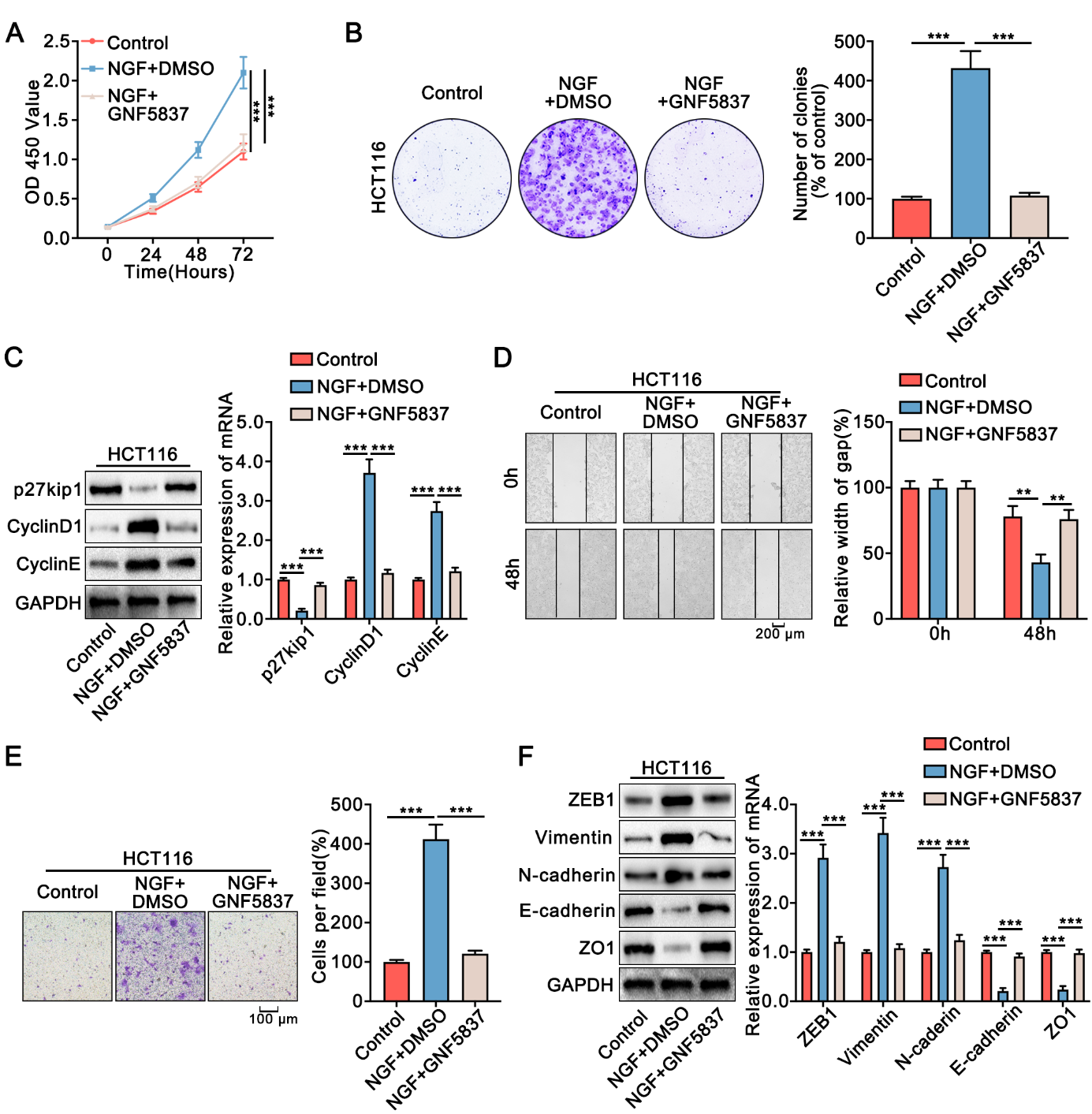
**

**Figure S8.** **NGF modulated the proliferation and metastasis of colon cancer cells by TrkA.** **(A).** CCK8 assay showed that inhibition of TrkA reversed the enhanced proliferation of HCT116 cells upon administration of NGF. **(B).** Colony formation assay indicated that inhibition of TrkA reversed the increased number and size of the colony upon administration of NGF. **(C).** Western blot and qRT-PCR showed that inhibition of TrkA blocked the altered expression of p27kip1, CyclinD1, and CyclinE in HCT116 cells caused by NGF. **(D).** The wound healing assays indicated that inhibition of TrkA significantly reversed the strengthened migrative ability of HCT116 cells upon administration of NGF. **(E).** The transwell assays indicated that inhibition of TrkA reversed the strengthened migrative ability of HCT116 cells upon administration of NGF. **(F).** Western blot and qRT-PCR showed that inhibition of TrkA blocked the altered expression of ZEB1, Vimentin, N-cadherin, E-cadherin, and ZO1 upon administration of NGF. DMSO was used as a control. GNF5837: the inhibitor of TrkA. All data were revealed as means ± standard deviation (SD) for no less than three independent experiments. Significant *P* values showed as ****P <* 0.001. n.s means the difference was not significant.

**
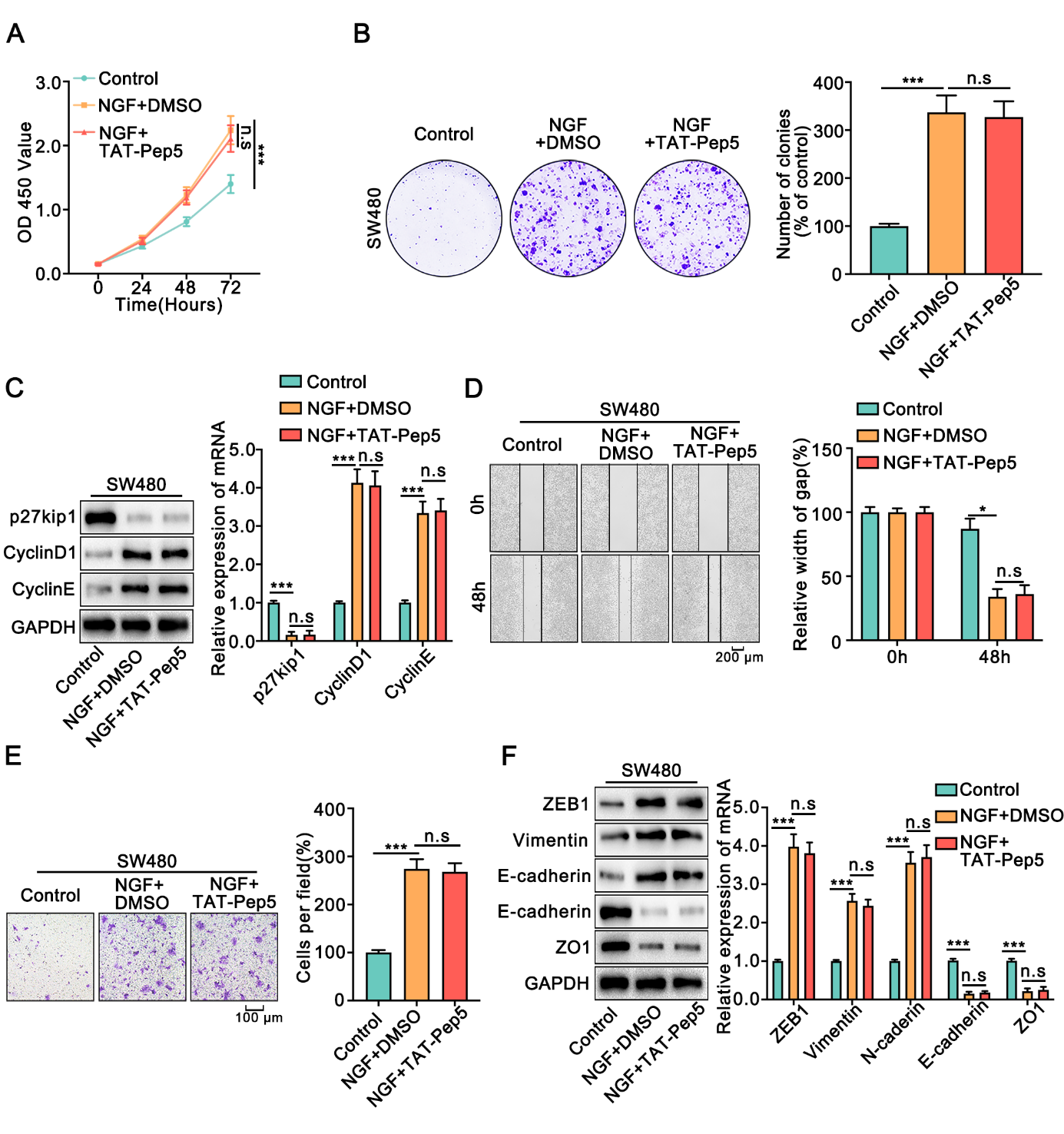
**

**Figure S9. P75 did not involve in the NGF-induced proliferation and metastasis of colon cancer cells.** **(A).** CCK8 assay showed that TAT-Pep5 did not affect the enhanced proliferation of SW480 cells upon administration of NGF. **(B).** Colony formation assay indicated that TAT-Pep5 did not affect the increased number and size of the colony upon administration of NGF. **(C).** Western blot and qRT-PCR showed that TAT-Pep5 did not affect the altered expression of p27kip1, CyclinD1, and CyclinE in SW480 cells caused by NGF. **(D).** The wound healing assays indicated that TAT-Pep5 did not affect the strengthened migrative ability of SW480 cells upon administration of NGF. **(E).** The transwell assays indicated that TAT-Pep5 did not affect the strengthened migrative ability of SW480 cells upon administration of NGF. **(F).** Western blot and qRT-PCR showed that TAT-Pep5 did not affect the altered expression of ZEB1, Vimentin, N-cadherin, E-cadherin, and ZO1 in SW480 cells upon administration of NGF. DMSO was used as a control. TAT-Pep5: the inhibitor of p75. All data were revealed as means ± standard deviation (SD) for no less than three independent experiments. Significant *P* values showed as ****P <* 0.001. n.s means the difference was not significant.

**
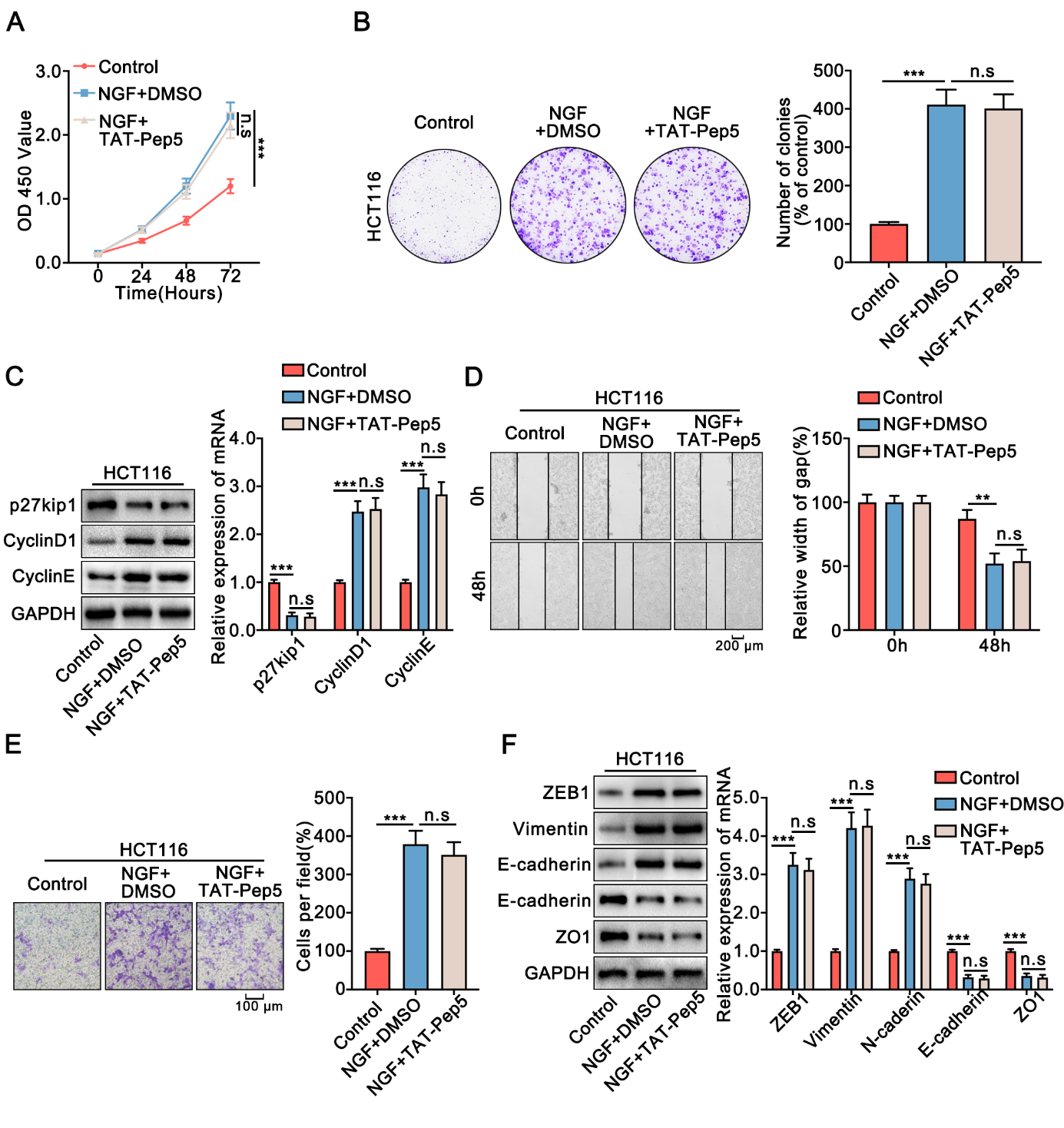
**

**Figure S10. P75 did not involve in the NGF-induced proliferation and metastasis of colon cancer cells. (A).** CCK8 assay showed that TAT-Pep5 did not affect the enhanced proliferation of HCT116 cells upon administration of NGF. **(B).** Colony formation assay indicated that TAT-Pep5 did not affect the increased number and size of the colony upon administration of NGF. **(C).** Western blot and qRT-PCR showed that TAT-Pep5 did not affect the altered expression of p27kip1, CyclinD1, and CyclinE in HCT116 cells caused by NGF. **(D).** The wound healing assays indicated that TAT-Pep5 did not affect the strengthened migrative ability of HCT116 cells upon administration of NGF. **(E).** The transwell assays indicated that TAT-Pep5 did not affect the strengthened migrative ability of HCT116 cells upon administration of NGF. **(F).** Western blot and qRT-PCR showed that TAT-Pep5 did not affect the altered expression of ZEB1, Vimentin, N-cadherin, E-cadherin, and ZO1 in HCT116 cells upon administration of NGF. DMSO was used as a control. TAT-Pep5: the inhibitor of p75. All data were revealed as means ± standard deviation (SD) for no less than three independent experiments. Significant *P* values showed as ****P <* 0.001. n.s means the difference was not significant.

**
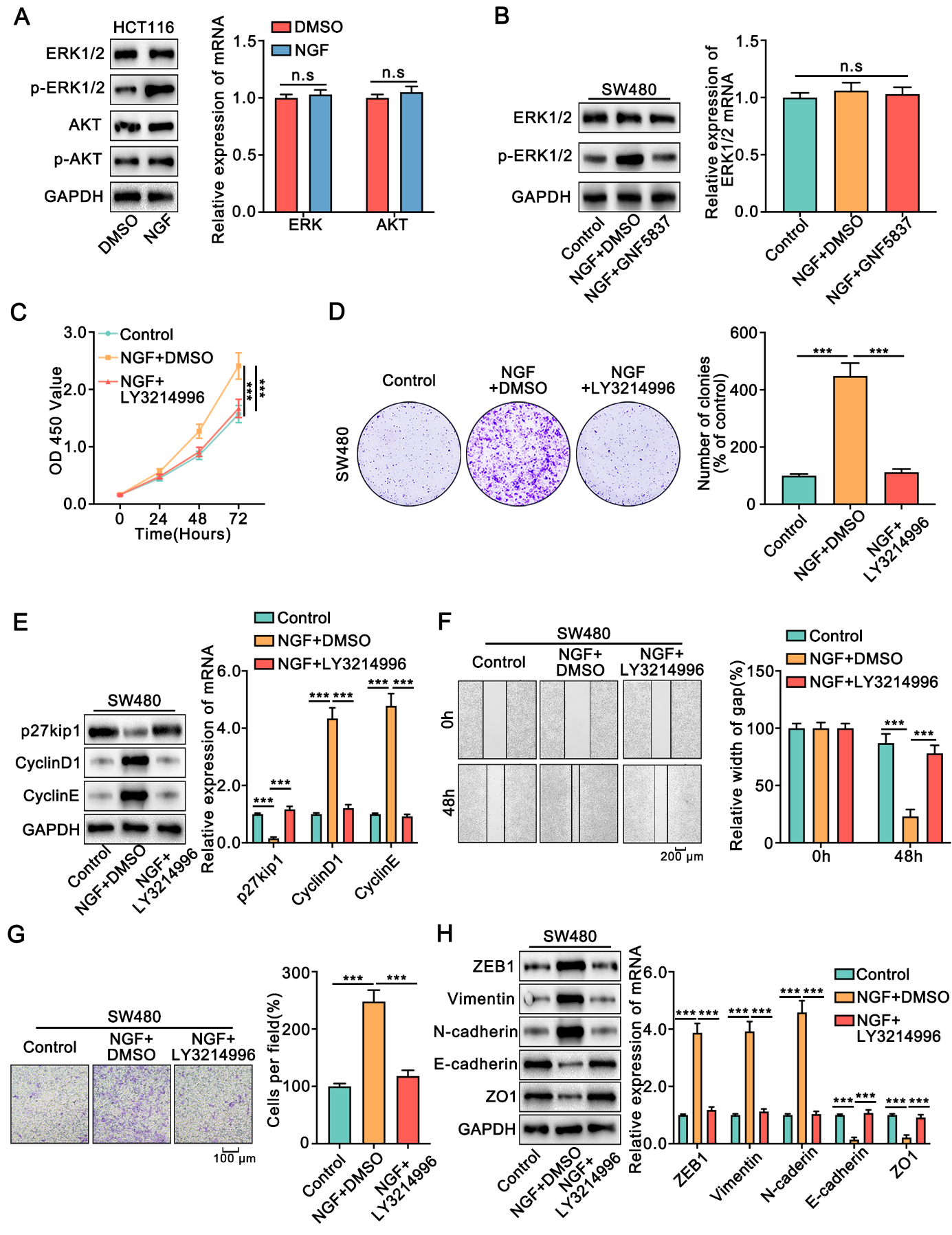
**

**Figure S11. NGF modulated the proliferation and metastasis of colon cancer cells through ERK. (A).** The expression of ERK1/2, phosphorylated ERK1/2, AKT, and phosphorylated AKT in HCT116 cells upon administration of NGF were detected by Western blot and qRT-PCR. **(B).** Western blot and qRT-PCR showed that inhibition of TrkA blocked the increased expression of phosphorylated ERK1/2 in SW480 upon administration of NGF. **(C).** CCK8 assay showed that LY3214996 reversed the enhanced proliferation of SW480 cells upon overexpression of NGF. **(D).** Colony formation assay indicated that LY3214996 reversed the increased number and size of the colony upon administration of NGF. **(E).** Western blot and qRT-PCR showed that LY3214996 blocked the altered expression of p27kip1, CyclinD1, and CyclinE in SW480 cells caused by NGF. **(F).** The wound healing assays indicated that LY3214996 significantly reversed the strengthened migrative ability of SW480 cells upon administration of NGF. **(G).** The transwell assays indicated that LY3214996 reversed the strengthened migrative ability of SW480 cells upon administration of NGF. **(H).** Western blot and qRT-PCR showed that LY3214996 blocked the altered expression of ZEB1, Vimentin, N-cadherin, E-cadherin, and ZO1 upon administration of NGF. DMSO was used as a control. GNF5837: the inhibitor of TrkA. LY3214996: the inhibitor of ERK. All data were revealed as means ± standard deviation (SD) for no less than three independent experiments. Significant *P* values showed as ****P <* 0.001.

**
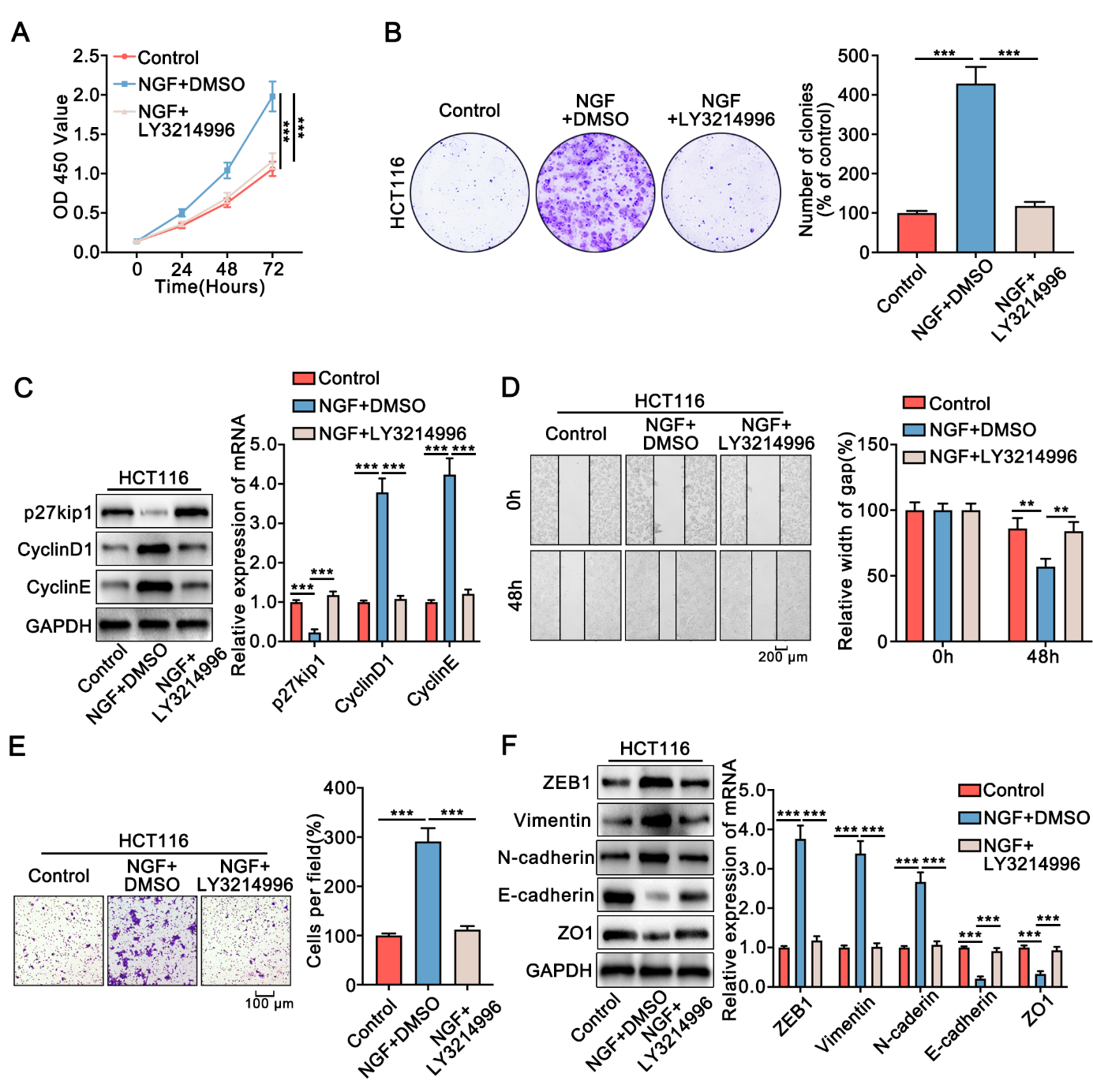
**

**Figure S12. NGF modulated the proliferation and metastasis of colon cancer cells through ERK. (A).** CCK8 assay showed that LY3214996 reversed the enhanced proliferation of HCT116 cells upon overexpression of NGF. **(B).** Colony formation assay indicated that LY3214996 reversed the increased number and size of the colony upon administration of NGF. **(C).** Western blot and qRT-PCR showed that LY3214996 blocked the altered expression of p27kip1, CyclinD1, and CyclinE in HCT116 cells caused by NGF. **(D).** The wound healing assays indicated that LY3214996 significantly reversed the strengthened migrative ability of HCT116 cells upon administration of NGF. **(E).** The transwell assays indicated that LY3214996 reversed the strengthened migrative ability of HCT116 cells upon administration of NGF. **(F).** Western blot and qRT-PCR showed that LY3214996 blocked the altered expression of ZEB1, Vimentin, N-cadherin, E-cadherin, and ZO1 upon administration of NGF. DMSO was used as a control. LY3214996: the inhibitor of ERK. All data were revealed as means ± standard deviation (SD) for no less than three independent experiments. Significant *P* values showed as ****P <* 0.001.

**
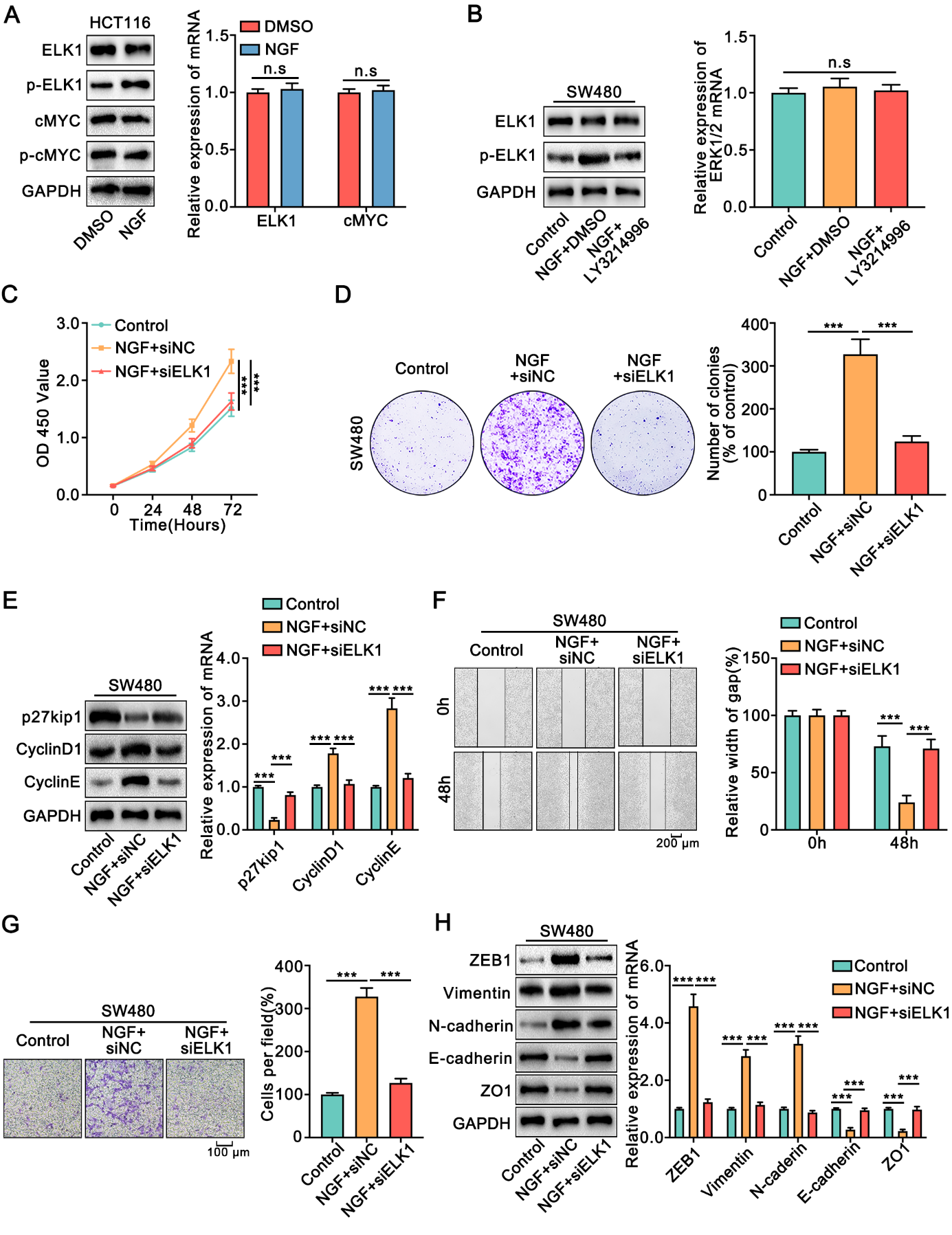
**

**Figure S13. NGF modulated the proliferation and metastasis of colon cancer cells via ERK/ELK1. (A).** The expression of ELK1, phosphorylated ELK1, cMYC, and phosphorylated cMYC in HCT116 cells upon administration of NGF were detected by Western blot and qRT-PCR. **(B).** Western blot and qRT-PCR showed that inhibition of ERK blocked the increased expression of phosphorylated ELK1 in SW480 upon administration of NGF. **(C).** CCK8 assay showed that knockdown of ELK1 reversed the enhanced proliferation of SW480 cells upon administration of NGF. **(D).** Colony formation assay indicated that knockdown of ELK1 reversed the increased number and size of the colony upon administration of NGF. **(E).** Western blot and qRT-PCR showed that knockdown of ELK1 blocked the altered expression of p27kip1, CyclinD1, and CyclinE in SW480 cells caused by NGF. **(F).** The wound healing assays indicated that knockdown of ELK1 significantly reversed the strengthened migrative ability of SW480 cells upon administration of NGF. **(G).** The transwell assays indicated that the knockdown of ELK1 reversed the strengthened migrative ability of SW480 cells upon administration of NGF. **(H).** Western blot and qRT-PCR showed that knockdown of ELK1 blocked the altered expression of ZEB1, Vimentin, N-cadherin, E-cadherin, and ZO1 upon overexpression of NGF. DMSO was used as a control. LY3214996: the inhibitor of ERK. All data were revealed as means ± standard deviation (SD) for no less than three independent experiments. Significant *P* values showed as ****P <* 0.001.


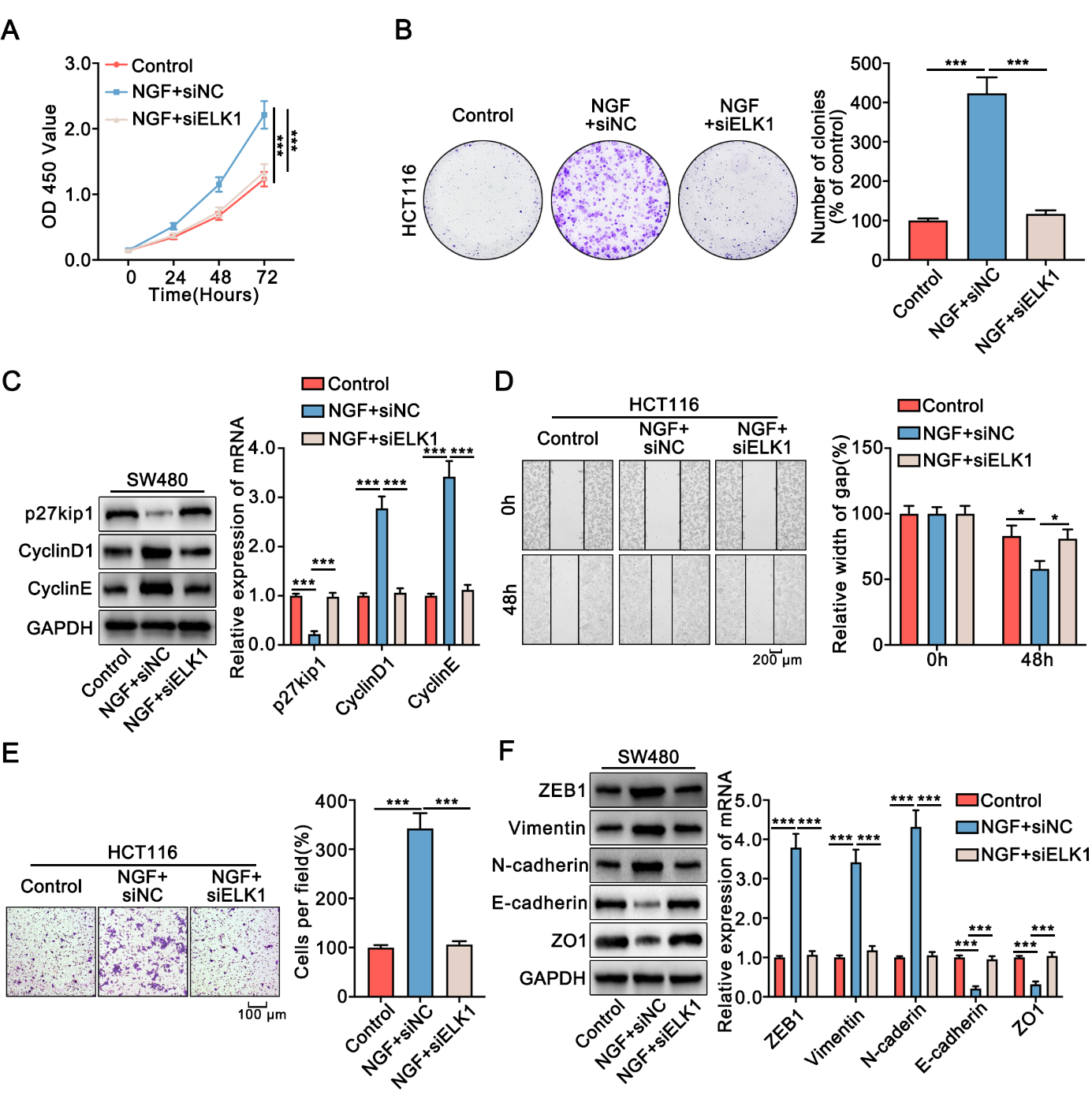


**Figure S14. NGF modulated the proliferation and metastasis of colon cancer cells via ERK/ELK1. (A).** CCK8 assay showed that knockdown of ELK1 reversed the enhanced proliferation of HCT116 cells upon administration of NGF. **(B).** Colony formation assay indicated that knockdown of ELK1 reversed the increased number and size of the colony upon administration of NGF. **(C).** Western blot and qRT-PCR showed that knockdown of ELK1 blocked the altered expression of p27kip1, CyclinD1, and CyclinE in HCT116 cells caused by NGF. **(D).** The wound healing assays indicated that knockdown of ELK1 significantly reversed the strengthened migrative ability of HCT116 cells upon administration of NGF. **(E).** The transwell assays indicated that the knockdown of ELK1 reversed the strengthened migrative ability of HCT116 cells upon administration of NGF. **(F).** Western blot and qRT-PCR showed that knockdown of ELK1 blocked the altered expression of ZEB1, Vimentin, N-cadherin, E-cadherin, and ZO1 upon overexpression of NGF. DMSO was used as a control. All data were revealed as means ± standard deviation (SD) for no less than three independent experiments. Significant *P* values showed as ****P <* 0.001.


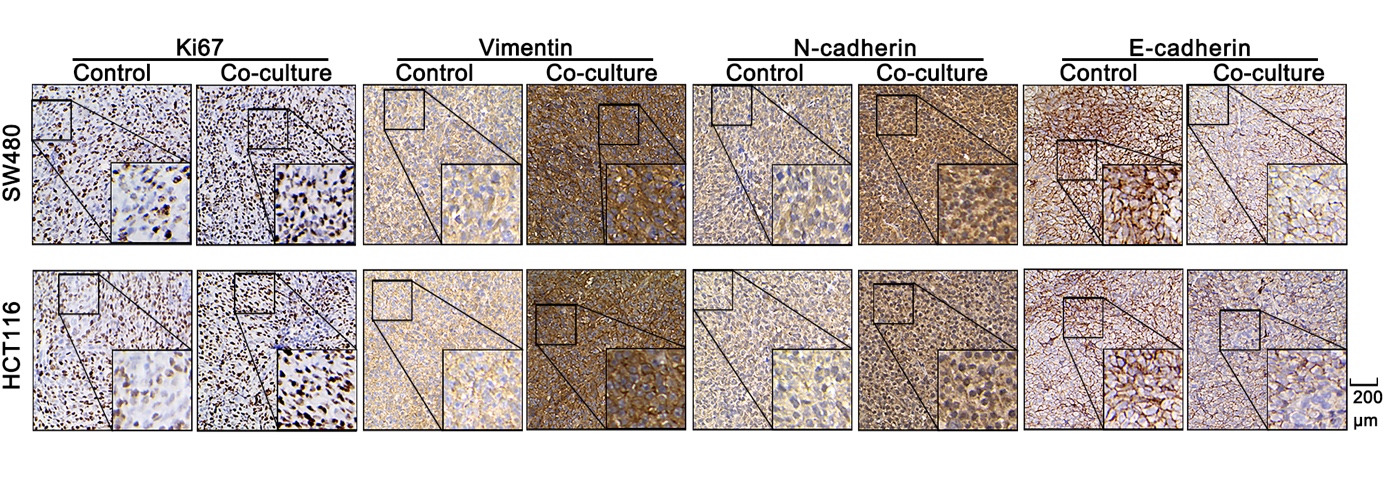


**Figure S15.** **Schwann cells accelerated the tumorigenesis and metastasis of colon cancer *in vivo*.** The expression of MIB1 (Ki 67), Vimentin, N-cadherin, and E-cadherin were detected in subcutaneous tumors’ tissues by IHC. All data were revealed as means ± standard deviation (SD) for no less than three independent experiments. Significant *P* values showed as ***P* < 0.01, and ****P <* 0.001.


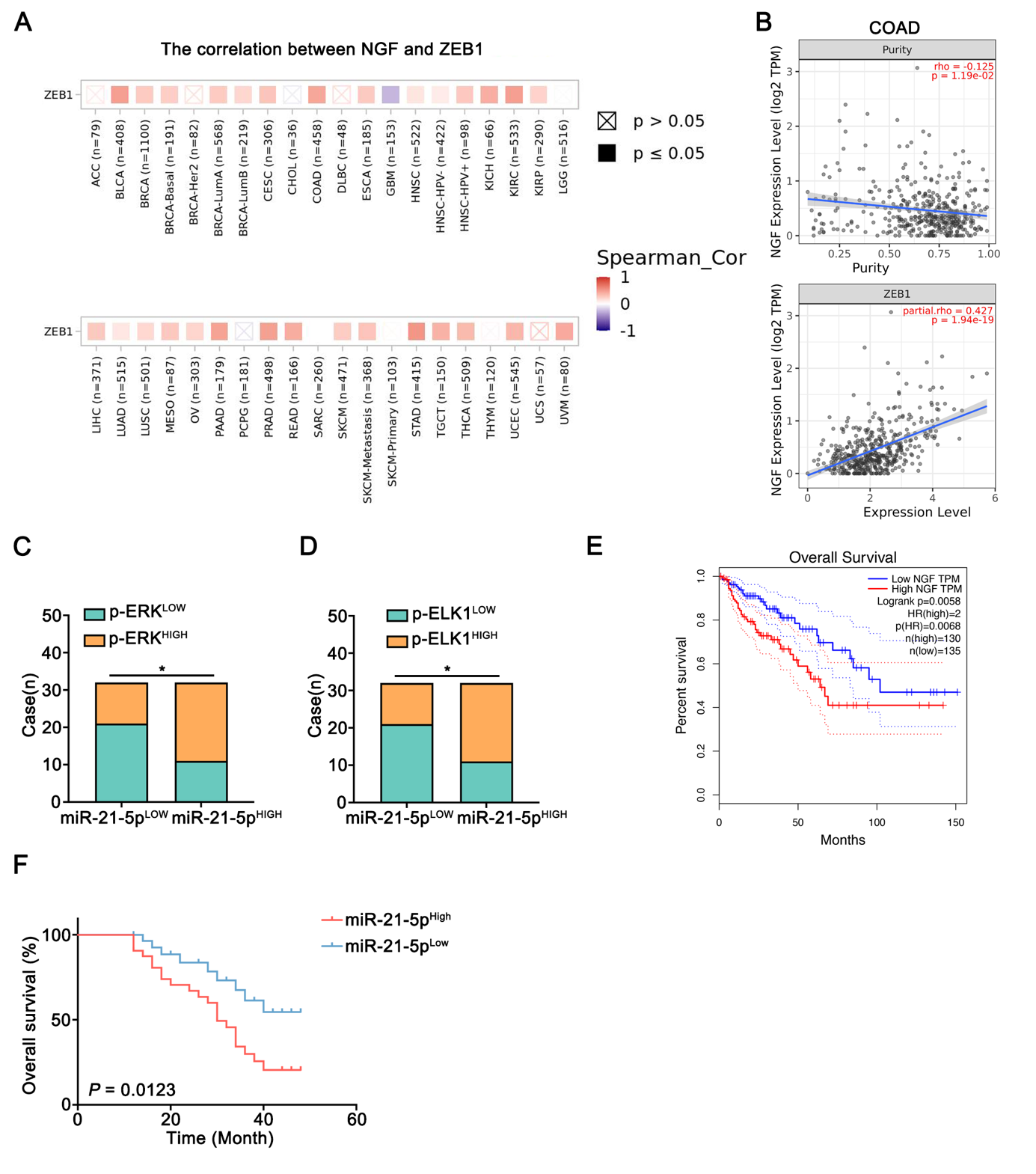


**Figure S16.** **The associated expression of NGF/TrkA/ERK/ELK1/ZEB1/miR-21-5p signaling in colon cancer tissues (A, B)** TIMER2.0 showed purity-adjusted correlation between NGF and ZEB1 in various cancer types (A) and COAD, exclusively (B). **(C, D).** The *chi*-square test identified a positive correlation between miR-21-5p and p-ERK and p-ELK1 in the colon cancer specimens. **(E).** Kaplan-Meier survival analysis indicated that the CRC patients in the NGF-HIGH group had a shorter overall survival time from GEPIA database. **(F)** Kaplan-Meier survival analysis indicated that the CRC patients in the miR-21-5p-HIGH group had a shorter overall survival time. Significant *P* values showed as **P <* 0.05.

**Supplementary methods**

**Cell culture and reagents**

Immortalized human colonic FHC cells, human colon cancer cells (HCT116 and SW480), and human Schwann cells sNF96.2 were purchased from American Type Culture Collection (ATCC, USA). FHC cells were cultured in DMEM/F12 (Gibco, USA). HCT116, SW480, and sNF96.2 cells were cultured in DMEM/high glucose (Gibco, USA). All cells were cultured in a medium supplemented with 10% fetal bovine serum (Gibco, USA) at 37°C under 5% CO_2_. The drugs used in this study included: DMSO (Sigma-Aldrich, USA), Human pro-beta-NGF (MCE, USA), Ro 08-2750: Inhibitor of NGF (MCE, USA), GNF5837: Inhibitor of TrkA (Selleck, USA), TAT-Pep5: Inhibitor of p75 (Merck, USA), and LY3214996: Inhibitor of ERK (Selleck, Texas, USA).

**CCK8 and EDU assays**

4 × 10^3^ cells/well were seeded on 96-well plates. At 0, 24, 48, and 72 hours, 10 μL CCK8 reagent (Dojondo Laboratories, Japan) was added after replacing the original medium and the plate was incubated for another two hours. Finally, the absorbance at 450 nm was measured by a microplate reader. EdU assay was performed using the EdU kit (RiboBio, China). 1 × 10^5^ cells/well were plated on 96-well plates overnight and incubated with 50 μM EdU for another two hours at 37°C. The nuclei were stained using 4’, 6-diamidino-2-phenylindole (DAPI; Sigma-Aldrich, USA) at room temperature for 30 minutes. Then, the EdU-positive cells were measured using fluorescence microscopy. All the assays were repeated 3 times with five technical replicates.

**Dual luciferase reporter assay for miRNA binding to 3’UTR**

To confirm whether VHL was a target of miR-21-5p, 1 × 10^5^ sNF96.2 cells were seeded on 96-well plates and cotransfected with dual luciferase reporter plasmid containing VHL 3’UTR (pGL3-VHL-3’UTR) or mutated forms with or without 50 nM miR-21-5p mimic or mimic-NC. After incubation for 48 hours, cells were lysed with diluted Passive Lysis Buffer. Luciferase Assay Buffer II was added and used to measure firefly luciferase activity. After stopping the reaction with 1XStop&Glo® Reagent, we measured the Renilla luciferase activity. Activity per well = firefly luciferase activity / Renilla luciferase activity.

**Dual luciferase reporter assay for transcription factor binding to promoter**

To investigate whether NGF is transcriptionally regulated by HIF-1α, 1 × 10^5^ sNF96.2 cells were seeded on 96-well plates and transfected with dual luciferase reporter plasmid containing NGF promoter (WT or MUT), and further co-transfected with siHIF-1α or siNC. After incubation for 48 hours, cells were lysed with diluted Passive Lysis Buffer. Luciferase Assay Buffer II was added and used to measure firefly luciferase activity. After stopping the reaction with 1XStop&Glo® Reagent, we measured the Renilla luciferase activity. Promoter activity per well = firefly luciferase activity / Renilla luciferase activity.

To examine whether ZEB1 is transcriptionally regulated by ELK1, 1 × 10^5^ HCT116 cells or SW480 cells were seeded on 96-well plates and transfected with dual luciferase reporter plasmid containing ZEB1 promoter (WT or MUT), and further co-transfected with siELK1 or siNC. The reporter activity was measured as above.

**Colony formation assay**

SW480 and HCT116 cells were cultured in 6-well plates with 500 cells per well and allowed to grow for 14 days in the recommended growth medium. The old medium was replaced with fresh medium every 3 days. Then, the clones were fixed with 4% paraformaldehyde for 20 minutes and stained with 0.1% crystal violet for 15 minutes. Finally, five fields of vision were chosen and the total number of colonies were counted to evaluate the results.

**Immunofluorescence analysis of the human specimens**

Tissues were formalin-fixed, dehydrated, paraffin-embedded and deparaffinized, and hydrated in graded alcohol. Then antigen retrieval procedure was performed in 10 nM sodium citrate buffer for 15 minutes, permeabilized in 0.5% Triton X-100 for 10 minutes, blocked in 5% donkey serum for 1 hour, incubated with the primary antibodies against GFAP (1:100, Abcam, USA) and S100B (1:100, Abcam, USA) overnight at 4 °C. Goat anti-mouse FITC (1:400, Abcam, USA) and donkey anti-rabbit Alexa Fluor 647 (1:400, Thermo, USA) were as the secondary antibodies. Finally, we captured the fluorescent images with epifluorescence microscope at 200×magnification.

**Chromatin immunoprecipitation (ChIP)**

We used EZ-ChIPTM Chromatin Immunoprecipitation Kit (Millipore, MA, USA) to perform the ChIP assay according to the manufacturer’s instructions. Briefly, cells were cross-linked with 1% formaldehyde for 10 min and then lysed on ice according to the instructions in the kit. The DNA was sheared with ultrasound. Antibodies and agarose beads were added to immunoprecipitate the cross-linked proteins and DNA. Rabbit anti-ELK1 (Abcam, Cambridge, UK), Rabbit anti- HIF-1α (Proteintech, IL, USA), and corresponding rabbit-IgG (CST, MA, USA) antibodies were used. Then, the protein and DNA complex were eluted and purified. After reverse cross-linking with 5M NaCl, the DNA was purified. The combined DNA fragments were amplified through qRT-PCR reactions, and the products were analyzed by 2% agarose gel electrophoresis.

**Bioinformatics analysis**

The potential binding sites were predicted from the JASPAR database (<https://jaspar.genereg.net/>). The miRNA sequencing data of colon cancer exosomes were downloaded from the GSE39833 dataset (https://www.ncbi.nlm.nih.gov/geo/). The miRNA data of colon cancer exosomes were searched in the EVmiRNA database (http://bioinfo.life.hust.edu.cn/EVmiRNA#!/browse). TargetScan database (https://www.targetscan.org/vert_80/) was applied to predict the potential target of miR-21-5p. The expression of NGF and ZEB1 in the TCGA cohort was downloaded from the GEPIA database (http://gepia.cancer-pku.cn/) and TIMER2.0 (http://timer.cistrome.org/).

**Animal experiments**

Male BALB/C nu/nu mice (5 weeks old) were purchased from Beijing Biotechnology Co. Ltd. (Beijing, China). A total of 5×10^6^ colon cancer cells were suspended in 120 μL PBS and injected into mice subcutaneously. We used calipers to measure tumor sizes every 7 days. On the twenty-eighth day, all the nude mice were sacrificed. Tumor size was measured using the following formula: V = 0.5 × (length) × (width)^2^. We injected approximately 6×10^6^ stable colon cancer cells into the tail vein of nude mice and evaluated pulmonary metastasis. Xenograft tumors were fixed with 4% paraformaldehyde for HE staining and IHC. Ro 08-2750 was administrated in p.o.at 100mg/kg/d. The stable inhibitor of miR-21-5p was purchased from GeneCopoeia (Maryland, USA). The above experimental animals were housed under standard conditions and processed following the National Research Council's animal care guidelines. The care and handling of the mice were approved by the Institutional Animal Care and Use Committee of Tongji Medical College, Huazhong University of Science and Technology.

**Table S1 Clinicopathological characteristics of colon cancer patients**

| Clinic pathological factors |  | GFAP^+^/S100B^+^ | | *P* value |
| --- | --- | --- | --- | --- |
|  | n | High | Low |  |
| Age(y) | | | | |
| ＞60 | 29 | 14 | 15 | 0.999 |
| ≤60 | 35 | 18 | 17 |  |
| Gender | | | | |
| Male | 34 | 15 | 19 | 0.452 |
| Female | 30 | 17 | 13 |  |
| Pathologic T stage | | | | |
| T1+T2 | 25 | 6 | 19 | 0.002 |
| T3+T4 | 39 | 26 | 13 |  |
| Pathologic N stage | | | | |
| N0 | 17 | 3 | 14 | 0.005 |
| N1+N2 | 47 | 29 | 18 |  |
| Pathologic M stage | | | | |
| M0 | 25 | 7 | 18 | 0.010 |
| M1 | 39 | 25 | 14 |  |
| Tumor differentiation | | | | |
| Well | 12 | 5 | 7 | 0.709 |
| Moderate | 23 | 11 | 12 |  |
| Poor | 29 | 16 | 13 |  |

Clinicopathological characteristics of patients in Wuhan Concorde hospital between 2015 and 2018. None of the patients received chemoradiotherapy before surgery. The data was tested by χ2 analysis. P < 0.05 was considered to be statistically significant.

**Table S2 Primers of genes in this research for qRT‐PCR**

| **Primer set** | **Primers** | **Sequence (5’-3’)** |
| --- | --- | --- |
| **GAPDH** | Forward | GGGGAGCCAAAAGGGTCATCATCT |
|  | Reverse | GACGCCTGCTTCACCACCTTCTTG |
| **P27kip1** | Forward | AACGTGCGAGTGTCTAACGG |
|  | Reverse | CCCTCTAGGGGTTTGTGATTCT |
| **Cyclin D1** | Forward | GAACACGGCTCACGCTTAC |
|  | Reverse | CCCAGACCCTCAGACTTGC |
| **Cyclin E** | Forward | AAGGAGCGGGACACCATGA |
|  | Reverse | ACGGTCACGTTTGCCTTCC |
| **Vimentin** | Forward | GCTTCAGAGAGAGGAAGCCGAAAA |
|  | Reverse | CCGTGAGGTCAGGCTTGGAAA |
| **ZEB1** | Forward | GATGATGAATGCGAGTCAGATGC |
|  | Reverse | ACAGCAGTGTCTTGTTGTTGT |
| **N-cadherin** | Forward | TCAGGCGTCTGTAGAGGCTT |
|  | Reverse | ATGCACATCCTTCGATAAGACTG |
| **E-cadherin** | Forward | GCCCTGCCAATCCCGATGAAA |
|  | Reverse | GGGGTCAGTATCAGCCGCT |
| **ZO-1** | Forward | CTGGTGAAATCCCGGAAAAATGA |
|  | Reverse | TTGCTGCCAAACTATCTTGTGA |
| **NGF** | Forward | GGCAGACCCGCAACATTACT |
|  | Reverse | CACCACCGACCTCGAAGTC |
| **TrkA** | Forward | CCATCGTGAAGAGTGGTCTC |
|  | Reverse | GGTGACATTGGCCAGGGTCA |
| **P75** | Forward | CCTACGGCTACTACCAGGATG |
|  | Reverse | CACACGGTGTTCTGCTTGT |
| **ERK** | Forward | TACACCAACCTCTCGTACATCG |
|  | Reverse | CATGTCTGAAGCGCAGTAAGATT |
| **AKT** | Forward | TCCTCCTCAAGAATGATGGCA |
|  | Reverse | GTGCGTTCGATGACAGTGGT |
| **ELK1** | Forward | TCCCTGCTTCCTACGCATACA |
|  | Reverse | GCTGCCACTGGATGGAAACT |
| **cMYC** | Forward | GGCTCCTGGCAAAAGGTCA |
|  | Reverse | CTGCGTAGTTGTGCTGATGT |
| **VHL** | Forward | GCAGGCGTCGAAGAGTACG |
|  | Reverse | CGGACTGCGATTGCAGAAGA |
| **HIF-1α** | Forward | GAACGTCGAAAAGAAAAGTCTCG |
|  | Reverse | CCTTATCAAGATGCGAACTCACA |
| **U6** | Forward | CTCGCTTCGGCAGCACA |
|  | Reverse | AACGCTTCACGAATTTGCGT |
| **MiR-21-5p** | Forward | CGTCCTAGCTTATCAGACTGA |
|  | Reverse | GCAGGGTCCGAGGTATTC |
| **MiR-23a-5p** | Forward | UUCUCCGAACGUGUCACGUTT |
|  | Reverse | ACGUGACACGUUCGGAGAATT |
| **MiR-10b-5p** | Forward | CGCCTGCTTGGTAACCCTGACC |
|  | Reverse | GGGTCCCACCCAGAGTGAGGT |
| **MiR-10a-5p** | Forward | ACACTCCAGCTGGGTACCCTGTAGATCCGAA |
|  | Reverse | CTCAACTGGTGTCGTGGAGTCGGCAATTCAGTTGAGCACAAATT |
| **MiR-27b-3p** | Forward | TTATGCCCAGCGATGACC |
|  | Reverse | GGCTCCAACTTAACTGTCCC |

These primers sequences had been validated in the NCBI database.

**Table S3 details of primary antibodies applied in this study**

| Gene  Specificity | Manufacture of primary antibody | Catalogue number | Dilution rate | | specificity |
| --- | --- | --- | --- | --- | --- |
|  |  |  | WB | IF |  |
| P27kip1 | Proteintech | 67355-1-Ig | 1:1000 | - | Mouse |
| Cyclin D1 | Proteintech | 60186-1-Ig | 1:1000 | - | Mouse |
| Cyclin E | Proteintech | 11554-1-AP | 1:1000 | - | Rabbit |
| GAPDH | Proteintech | 60004-1-Ig | 1:1000 |  | Mouse |
| ZEB1 | Proteintech | 21544-1-AP | 1:1000 | - | Rabbit |
| Vimentin | Proteintech | 10366-1-AP | 1:1000 | 1:100 | Rabbit |
| ZO-1 | Proteintech | 66452-1-Ig | 1:1000 | - | Mouse |
| N-cadherin | Proteintech | 22018-1-AP | 1:1000 | 1:100 | Rabbit |
| E-cadherin | Proteintech | 20874-1-AP | 1:500 | 1:100 | Rabbit |
| NGF | Cell Signaling Technology | 2046S | 1:1000 | - | Rabbit |
| TrkA | Cell Signaling Technology | 2510S | 1:1000 | - | Rabbit |
| p-TrkA | Cell Signaling Technology | 4619S | 1:1000 | - | Rabbit |
| P75 | Proteintech | 55014-1-AP | 1:500 |  | Rabbit |
| ERK1/2 | Proteintech | 16433-1-AP | 1:1000 |  | Rabbit |
| p-ERK1/2 | Proteintech | 28733-1-AP | 1:500 |  | Rabbit |
| AKT | Proteintech | 60203-2-Ig | 1:1000 |  | Mouse |
| p-AKT | Proteintech | 66444-1-Ig | 1:500 |  | Mouse |
| ELK1 | Proteintech | 27420-1-AP | 1:1000 |  | Rabbit |
| p-ELK1 | Abcam | ab218133 | 1:500 |  | Rabbit |
| cMYC | Proteintech | 10828-1-AP | 1:1000 |  | Rabbit |
| p-cMYC | Abcam | ab185656 | 1:500 |  | Rabbit |
| VHL | Cell Signaling Technology | 68547S | 1:1000 |  | Rabbit |
| HIF-1α | Proteintech | 20960-1-AP | 1:1000 |  | Rabbit |
